# Expression of *RSOsPR10* in rice roots is antagonistically regulated by jasmonate/ethylene and salicylic acid via the activator OsERF87 and the repressor OsWRKY76, respectively

**DOI:** 10.1002/pld3.49

**Published:** 2018-03-30

**Authors:** Takahiro Yamamoto, Yuri Yoshida, Kazunari Nakajima, Makiko Tominaga, Atsuko Gyohda, Hiromi Suzuki, Takashi Okamoto, Takeshi Nishimura, Naoki Yokotani, Eiichi Minami, Yoko Nishizawa, Koji Miyamoto, Hisakazu Yamane, Kazunori Okada, Tomokazu Koshiba

**Affiliations:** ^1^ Department of Biological Sciences Tokyo Metropolitan University Hachioji‐shi Tokyo Japan; ^2^ Biotechnology Research Center The University of Tokyo Bunkyo‐ku Tokyo Japan; ^3^ Institute of Agrobiological Sciences National Agriculture and Food Research Organization Tsukuba Ibaraki Japan; ^4^ Bioagric Sci Nagoya University Nagoya Aichi Japan; ^5^ Kazusa DNA Research Institute Kisarazu Chiba Japan; ^6^ Department of Biosciences Teikyo University Utsunomiya Tochigi Japan

**Keywords:** jasmonic acid, MED25, OsERF87, OsWRKY76, pathogenesis‐related protein, rice root, RSOsPR10, salicylic acid

## Abstract

Plant roots play important roles in absorbing water and nutrients, and in tolerance against environmental stresses. Previously, we identified a rice root‐specific pathogenesis‐related protein (RSOsPR10) induced by drought, salt, and wounding. *RSOsPR10* expression is strongly induced by jasmonate (JA)/ethylene (ET), but suppressed by salicylic acid (SA). Here, we analyzed the promoter activity of *RSOsPR10*. Analyses of transgenic rice lines harboring different‐length *promoter::*β*‐glucuronidase* (*GUS*) constructs showed that the 3‐kb promoter region is indispensable for JA/ET induction, SA repression, and root‐specific expression. In the JA‐treated *3K‐promoter::GUS* line, GUS activity was mainly observed at lateral root primordia. Transient expression in roots using a dual luciferase (LUC) assay with different‐length *promoter::LUC* constructs demonstrated that the novel transcription factor OsERF87 induced *3K‐promoter::LUC* expression through binding to GCC‐*cis* elements. In contrast, the SA‐inducible OsWRKY76 transcription factor strongly repressed the JA‐inducible and OsERF87‐dependent expression of *RSOsPR10*. *RSOsPR10* was expressed at lower levels in *OsWRKY76*‐overexpressing rice, but at higher levels in *OsWRKY76*‐knockout rice, compared with wild type. These results show that two transcription factors, OsERF87 and OsWRKY76, antagonistically regulate *RSOsPR10* expression through binding to the same promoter. This mechanism represents a fine‐tuning system to sense the balance between JA/ET and SA signaling in plants under environmental stress.

## 1 INTRODUCTION

Plants are constantly exposed to various types of biotic stresses such as pathogen attack and insect herbivory, and abiotic stresses like wounding, drought, and high salinity. To survive these challenges, plants have developed elaborate mechanisms to perceive external signals and manifest adaptive responses with appropriate physiological changes. Some pathogenesis‐related (PR) proteins are known to be induced not only during biotic pathogen infection but also in response to abiotic stresses (Agarwal & Agarwal, 2014; van Loon, Rep, & Pieterse, 2006). As the major pollen allergen (Bet v1) from white birch was first identified as a PR10‐class protein, there have been many studies on PR10 proteins in diverse plant species (Agarwal & Agarwal, 2014; Liu & Ekramoddoullah, 2006). Ectopic overexpression of *PR10* from several plant sources has been shown to increase tolerance to various abiotic stresses, such as salt and drought (Huang, Lin, et al., 2016; Lee, Pulla, Kim, Balusamy, & Yang, 2012; Takeuchi et al., 2016). Interestingly, genetic, structural, and bioinformatic studies have revealed highly diverse functions of the PR10 family, such as cytokinin‐, flavonoid‐, and steroid‐binding activities (Fernandes et al., 2008; Zubini et al., 2009), membrane permeabilization activity (Mogensen et al., 2007), norcoclaurine synthase activity (Lee & Facchini, 2010), and abscisic acid (ABA) receptor activity (Ma et al., 2009). Therefore, further studies on the physiological and molecular functions of PR10 proteins and on the regulation of their encoding gene expression will be of increasing importance to improve the performance of plants under various environmental stresses.

Several studies have focused on the regulation of expression of defense‐response PR proteins by the levels of endogenous plant hormones such as salicylic acid (SA), jasmonate (JA), ethylene (ET), and their combinations/crosstalk (Koornneef & Pieterse, 2008; Thaler, Humphrey, & Whiteman, 2012). The SA signaling pathway is important for general defense responses and especially for attack by biotrophic pathogens, and the JA/ET signaling pathway is involved in responses to abiotic stresses as well as in defense signaling against necrotrophic pathogens. The crosstalk between SA and JA/ET in defense against biotic and abiotic stresses has been well studied and has considerable agricultural importance (Grant & Jones, 2009). One of the main factors in the SA pathway, nonexpressor of pathogenesis‐related genes 1 (NPR1), regulates trafficking between JA/ET and SA defense responses via transcription factors such as TGAs and WRKYs (Leon‐Reyes et al., 2009; Spoel et al., 2009; Zander, La Camera, Lamotte, Metraux, & Gatz, 2010). Other factors and complex mechanisms are involved in the antagonism between the JA/ET and SA pathways, and researchers have considered the role of these mechanisms in saving energy by controlling the allocation of resources depending on the types of attackers, because defenses are costly to produce (Ahmad et al., 2016; De Vleesschauwer, Gheysen, & Hofte, 2013; Thaler et al., 2012; Vos, Moritz, Pieterse, & Van Wees, 2015). The *Arabidopsis* defense‐related PR protein, PDF1.2 (PR12) is a good model of a JA/ET‐inducible and SA‐suppressible stress response protein, and several studies have explored the molecular mechanisms underlying the antagonistic regulation of *PDF1.2* expression (Koornneef et al., 2008). Factors in JA/ET and SA signaling pathways, like COI1, JAZs, NPR1, MYC2, TGAs, ERFs, and WRKYs, are known to be regulators of *PDF1.2* (Leon‐Reyes et al., 2009; Lorenzo, Piqueras, Sanchez‐Serrano, & Solano, 2003; McGrath et al., 2005; Van der Does et al., 2013; Zander, Thurow, & Gatz, 2014; Zarei et al., 2011). *Arabidopsis* ORA59/AtERF94 is an ERF transcription factor that induces *PDF1.2* via the JA/ET pathway through direct binding to GCC boxes in the *PDF1.2* promoter (Catinot et al., 2015; Huang, Catinot, & Zimmerli, 2016; Pre et al., 2008; Zarei et al., 2011). Group IXc ERF members in *Arabidopsis*, including ORA59, act as transcriptional activators by binding to the eukaryotic transcriptional complex (mediator complex) through the EDLL motif in the group‐IXc‐specific CMIX‐1 domain (Huang, Catinot, et al., 2016; Kazan, 2017). In contrast, only a few studies have reported on the antagonistic suppression by the SA pathway resulting in indirect repression upstream of ORA59 (Nakata et al., 2013; Van der Does et al., 2013; Zander et al., 2014). One possible explanation is that repressor‐type SA‐inducible ERF(s) (AtERF4 and AtERF9 in group VIII ERF) suppress *PDF1.2* through binding to GCC box(es) in its promoter (Caarls, Pieterse, & Van Wees, 2015; Maruyama et al., 2013; McGrath et al., 2005). However, Caarls et al. showed that repressor‐type ERFs are not essential for suppression of *PDF1.2* transcription, and proposed that some SA‐induced unknown protein may suppress JA‐induced transcription (Caarls et al., 2017). Several studies have proposed that SA‐mediated repressor‐type WRKY transcription factors may be candidates as direct suppressors of *PDF1.2* expression, but this has not been proven experimentally (Birkenbihl, Diezel, & Somssich, 2012; Caarls et al., 2015; Van der Does et al., 2013; Zander et al., 2014). Therefore, there is still much discussion about the possible mechanisms of the antagonistic regulation of *PDF1.2* expression by JA/ET and SA signaling.

In our previous studies, we showed that the expression of *RSOsPR10* in rice roots is induced by abiotic stresses such as drought, wounding, and high salt, and by the plant hormones JA and ET, while SA almost completely suppresses its induction (Hashimoto et al., 2004; Takeuchi et al., 2011). Transgenic rice and bentgrass lines overexpressing *RSOsPR10* exhibited increased tolerance to drought (rice and bentgrass) and salt (bentgrass) (Takeuchi et al., 2016). The results indicated a possible function of RSOsPR10 in promoting root growth and root mass, which could lead to increased tolerance to soil desiccation and high‐salt conditions. Therefore, it is important to resolve the function and role of RSOsPR10 in nature, and to determine how the expression of its encoding gene is regulated in response to environmental challenges. In the course of our research, we have observed the clear antagonistic regulation of *RSOsPR10* expression by JA/ET and SA pathways, similar to that of the *Arabidopsis* defense gene *PDF1.2*. Here, we focused on the transcriptional regulation of *RSOsPR10* expression, using rice lines harboring *RSOsPR10 promoter::GUS* constructs in which the promoter fragments ranged from 2 to 4 kb in length. The results showed that the 3‐kb upstream region is important for JA/ET induction, SA suppression, and root‐specific expression. To analyze *cis*‐acting elements and *trans*‐factors, we further performed promoter analyses using transient expression dual LUC reporter assays. The results indicated that the novel rice ERF transcription factor, OsERF87, an Arabidopsis ORA59 ortholog, participates in JA induction of *RSOsPR10* through binding to GCC boxes in the 3‐kb promoter region. While OsERF87 promoted *RSOsPR10* expression, the co‐expression of SA‐inducible OsWRKY76 clearly suppressed the induction of the 3‐kb promoter by OsERF87. The results of this study showed that two transcription factors, the activator OsERF87 and the repressor OsWRKY76, antagonistically regulate the expression of *RSOsPR10* through binding to its promoter. These results are the first clear evidence of a transcriptional‐level fine‐tuning mechanism regulating the expression of a defense‐related PR protein through sensing the balance between JA/ET and SA in rice roots.

## 2 METHODS

### 2.1 Plant materials and growth conditions

Rice (*Oryza sativa* L, cv. Nipponbare) seeds were grown on 0.3% agar medium for 4 days and then transferred onto a floating polyethylene board with holes approximately 5 mm in diameter. The seedlings were grown under the following controlled environment conditions: 27°C, 12‐h light (65.4 μmol m^−2^ s^−1^) /12‐h dark cycle (Takeuchi et al., 2011). Seeds of transgenic rice lines harboring different‐length *RSOsPR10 promoter::GUS* constructs and seeds of *OsWRKY76‐OX* and *OsWRKY76*‐*KO* mutants (background *O. sativa* L. cv. Nipponbare (Pia)) were germinated in the same conditions (Yokotani et al., 2013). The *OsWRKY76‐KO* line was produced using the *Agrobacterium*‐mediated gene‐targeting system based on homologous recombination (Ozawa et al., 2012). A 6.5‐kb fragment and a 5.8‐kb fragment corresponding to the upper and lower regions of the *OsWYKY76* locus, respectively, were amplified from rice total DNA by PCR using the primers as follows: W76‐UpAsc‐F1 (ACGGCGCGCCATTTTCTTATTCTCGAGGTTCTTTG), W76‐UpAsc‐R1 (CTGGCGCGCCGCTCTGGAGCTCGAGTAATCA), W76‐DwF1 (ATGCTGTACAGAATTCAGAAGCTGCCCGAATTCTAGCTTC), and W76‐DwR1 (TTACGCGTTTGAATTTGTGGTCTTCATTGTACATATCAAG). The two fragments were cloned into the binary vector for homologous recombination. The replacement of the *OsWRKY76* coding region with the HPT cassette was confirmed by genomic sequencing of the *OsWRKY76* locus. Seedlings (9 days old) were treated with JA and SA by dipping the roots into water containing 100 μM (+/−)‐JA (Sigma), 100 μM SA (WAKO), or a combination of these chemicals. In some experiments, seedlings were also treated with 100 μM ACC and 100 μM ACC + 100 μM SA. After treatments for appropriate time intervals, root samples, and, if appropriate, shoot samples (sheaths and leaves), were collected and frozen in liquid nitrogen.

### 2.2 Cloning of *RSOsPR10* promoter and generation of transgenic plants harboring *RSOsPR10 Promoter:Reporter* constructs

To generate *RSOsPR10 promoter::GUS* transgenic rice plants, the 4‐kb *RSOsPR10* promoter region upstream of the start codon was amplified by PrimeSTAR^®^ Max DNA Polymerase (TaKaRa Bio USA, Madison, WI, USA) using genomic DNA prepared from rice leaves as a template and specific primers (Table S2). The *RSOsPR10* promoter region was cloned into the pGEM‐T Easy Vector (Promega, Madison, WI, USA) to construct 4K‐pGEM. Three fragments (4087, 3005, or 1975 bp) of the upstream region of *RSOsPR10* were amplified, and each was inserted into the *Hin*dIII‐*Xba*I site of the pIG121‐Hm vector (Ohta, Mita, Hattori, & Nakamura, 1990) to construct *4K‐*,* 3K‐*, or *2K‐pBIH1*, using the In‐Fusion system (TaKaRa Bio USA). These constructs were transformed into scutellum‐derived calli of cv. Nipponbare using *Agrobacterium tumefaciens* LBA4404 Electro‐Cells (TaKaRa Bio USA) (Kiribuchi et al., 2004). Independent transgenic calli showing hygromycin and carbenicillin resistance were collected on selective solid medium, and T1 seeds were collected from regenerated transgenic plants. Genomic DNA was extracted from the transgenic plants using the DNeasy Plant Mini Kit (Qiagen, Hilden, Germany) and subjected to PCR analysis using the *GUS*‐specific primers 5′‐GTCCTGTAGAAACCCC‐3′ (forward) and 5′‐ CAACAGACGCGTGGTT‐3′ (reverse).

### 2.3 RNA extraction and quantitative real‐time PCR (qRT‐PCR)

Total RNA was extracted from rice roots or shoots using the RNeasy Plant Mini Kit (Qiagen) and used to synthesize cDNA with the High Capacity RNA‐to‐cDNA‐Kit (Applied Biosystems, Foster City, CA, USA) according to the manufacturer's protocol. The qRT‐PCR analyses were performed with a LightCycler 480 (Roche Diagnostics, Mannheim, Germany) using SYBR Green I Master mix and the primers listed in Table S2. The relative expression level of mRNA was determined using *ubiquitin* as the reference gene. All qRT‐PCR experiments were performed in biological triplicate and technical duplicate.

### 2.4 Histochemical localization of GUS expression in rice roots

The roots of 9‐day‐old seedlings of *RSOsPR10 2K‐, 3K‐, and 4K‐promoter::GUS* lines (*line #11, #14, and #15*, respectively) were treated with 100 μM JA for 12 h. To detect GUS activity, the roots were stained with 1 mM 5‐bromo‐4‐chloro‐3‐indolyl β‐D‐glucuronide (Wako) as described previously (Nishimura et al., 2014). The GUS‐stained roots and cross sections of roots fixed in 70% ethanol were observed under a stereomicroscope (M420; Leica) and a microscope (BX51, Olympus), respectively, each equipped with a digital camera (DP50; Olympus).

### 2.5 *Promoter:LUC* construction

Each of the 4,087‐, 3,395‐, 3,005‐, 2,782‐, 1,975‐bp upstream regions of the *RSOsPR10* promoter was PCR‐amplified using 4K‐pGEM as the template. Each fragment was inserted into the *Hin*dIII‐*Nco*I site of pGL4.10‐Tnos (Ogawa et al., 2017) using an In‐Fusion kit (TaKaRa Bio USA) to obtain 4K‐, 3.4K‐, 3K‐, 2.8K‐, or 2K‐pGL. The 0.3‐kb *Xba*I‐*Bam*HI fragment of pGL4.10‐Tnos including the nopaline synthase terminator (Tnos) region was inserted into the *Xba*I‐*Bam*HI site of the pRL‐null vector (Promega, Madison, WI, USA) to construct Tnos‐pRL. The 2.6‐kb upstream region of the rice *ubiquitin* promoter was PCR‐amplified and inserted into the Tnos‐pRL to obtain pUbi‐pRL, which was used as an internal control.

### 2.6 Construction of GCC‐ and W‐box mutations

Mutation constructs of GCC boxes (*3K*[G4 m]*, 3K*[G2‐5m]) and the W box (*3K*[W2 m]) were generated using the PrimeSTAR Mutagenesis Basal Kit (TaKaRa Bio USA) with the modification that PCR‐amplified DNA fragments were treated with *Dpn*I before insertion into *Escherichia coli*.

### 2.7 Particle Bombardment

To coat gold particles, 1 μg reporter plasmid and 0.5 μg internal control plasmid were precipitated onto 0.6‐mg gold particles (1.0 μm diameter) for each bombardment. Nine‐day‐old rice seedlings were placed on 1% agar (Wako 016‐11875) plates (3–4 seedlings/plate). Then, root tissue was bombarded twice at 1100 psi using the PDS‐1000 He Biolistic Particle Delivery System (Bio‐Rad; Hercules, CA, USA). After bombardment, the seedlings were kept on the plates for 30 min and then immersed in water containing JA and/or SA for 12 h at 27°C in darkness. After incubation, rice roots were homogenized in 200 μL cell lysis buffer (Promega). The homogenates were centrifuged, and the supernatants were used in the luciferase activity assay.

### 2.8 Luciferase gene reporter assay

The luciferase assay was performed using the dual luciferase reporter assay system (Promega) according to the manufacturer's instructions. Luminescence was measured using a luminometer (GLOMAX 20/20, Promega). The relative activity of firefly luciferase (FLUC)/renilla luciferase (RLUC) was recorded.

### 2.9 Effector assay

The *GUS* sequence in pBI221 was replaced with the coding sequence of *OsWRKY76*. The plasmid expressing the GAL4 DNA‐binding (DB) domain was used as a control effector (Yokotani et al., 2013). *OsERF1, OsERF83,* and *OsERF87* sequences were amplified from cDNAs from rice roots by PCR using Prime STAR GXL DNA Polymerase (TaKaRa Bio USA) with specific primers (Table S2). Amplified DNA fragments were purified with a QIA quick Gel Extraction Kit (Qiagen) according to the manufacturer's protocol. The *OsERF1* and *OsERF83* fragments were subcloned into the pGEM‐T Easy vector and then into pBI221 via *Sac*I and *Xba*I sites. The amplified *OsERF87* DNA fragment was subcloned into pDONR™/Zeo using GATEWAY Technology (Invitrogen, Carlsbad, CA, USA) and then recombined into pGWB502 (Nakagawa et al., 2007) by the LR reaction according to the manufacturer's protocol. *OsERF136* was amplified by PCR from cDNAs obtained from rice roots using KOD FX (Toyobo, Osaka, Japan) using specific primers. The *OsERF1*36 fragment was cloned into the *Sac*I‐*Xba*I site of pBI221 using an In‐Fusion HD Cloning Kit (Clontech, Palo Alto, CA, USA). For cotransfection assays, 1 μg reporter plasmid, 1 μg effector plasmid, and 0.5 μg internal control plasmid were introduced into roots of 9‐day‐old rice plants as described above.

### 2.10 Transient expression of GFP‐OsERF87 in onion epidermal cells

The *OsERF87* ORF was fused into pGW505 (Nakagawa et al., 2007) using LR Clonase II Enzyme Mix (Thermo Fisher Scientific). We used 35S:GFP (Chiu et al., 1996) as a control. The constructs were introduced into inner epidermis cells of onion bulb scales by particle bombardment as described above. After bombardment, the onion bulb scales were incubated in moist conditions overnight at 27°C in darkness. The fluorescence of GFP was observed using an Olympus IX71 microscope.

### 2.11 Gel mobility shift assay

The coding sequence of *OsWRKY76* was inserted between the *Bam*HI and *Hin*dIII sites of pMALc2x (New England Biolabs, Beverly, MA, USA) and introduced into *E. coli* JM109 as described previously (Yokotani et al., 2013). OsWRKY76 fused to maltose‐binding protein (MBP) was purified using amylose resin (New England Biolabs), according to the manufacturer's instructions. The nucleotide sequences of the probes used for DNA‐binding assays were as follows: WB (5′‐CTCCACCGCCTTGACCTATCGTCGCT‐3′), m1 (5′‐CTCCACCGCCTTGAaCTATCGTCGCT‐3′) and m2 (5′‐CTCCACCGCCTcctaCTATCGTCGCT‐3′) (W‐box and mutated W‐box core sequences underlined). Gel mobility shift assays were performed using the DIG Gel Shift Kit 2^nd^ Generation (Roche Diagnostics GmbH, Mannheim, Germany).

### 2.12 Accession numbers and gene IDs

Sequence data from this article can be found in the RiceXPro (http://ricexpro.dna.affrc.go.jp/category-select.php) and the Arabidopsis Information Resource (https://www.arabidopsis.org/) databases under the following accession numbers: OsERF1 (AK243188, Os04g46220/Os04g0546800), OsERF83 (AK109390, Os03g64260/Os03g0860100), OsERF87 (AK241281, Os09g39850/Os09g0572000), OsERF136 (AK108503, Os07g22730/Os07g0410300), OsWRKY28 (AK106282, Os06g44010/Os06g0649000), OsWRKY62 (AK067834, Os09g25070/Os09g0417800), OsWRKY76 (AK068337, Os09g25060/Os09g0417600), OsPR10b (AK070762, Os12g0555200), RSOsPR10 (AK241281, Os12g36830/Os12g0555000), ORA59/AtERF94 (BT014958, At1g06160), PDF1.2 (AY133787, At5g44420).

## 3 RESULTS

### 3.1 Identification of *RSOsPR10* promoter region responsible for JA/ET‐inducible, SA‐suppressible, and root‐specific expression using transgenic rice lines

**Figure 1:**
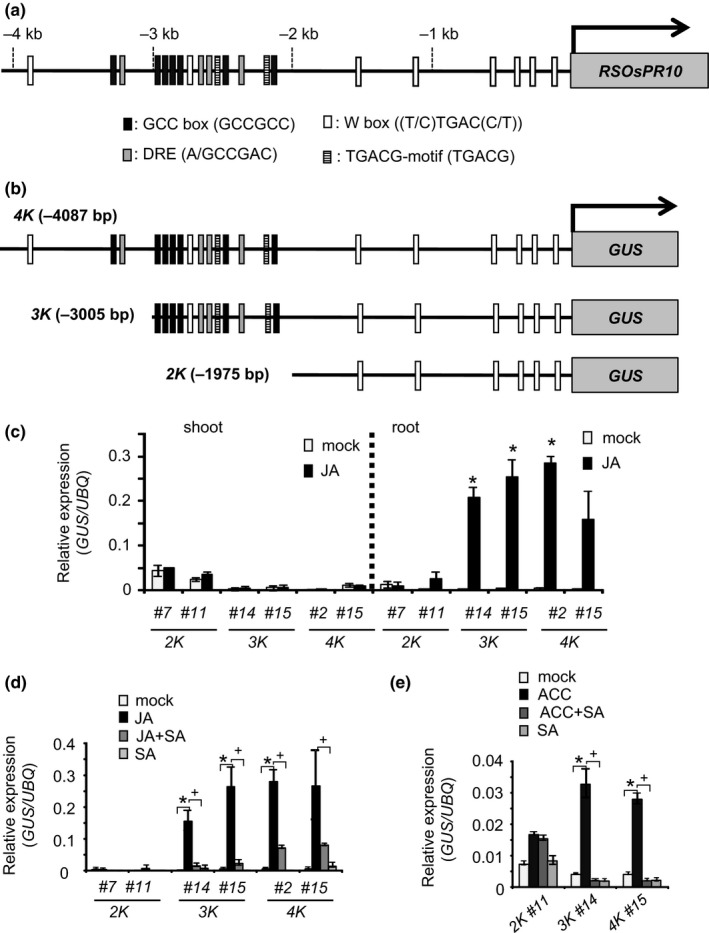
β‐Glucuronidase (*GUS*) mRNA levels in transgenic lines harboring different‐length *RSOsPR10 promoter::GUS* after treatment with plant hormones. (a) Database search of stress response‐related *cis* elements in *RSOsPR10* promoter (−1 to −4087 bp from translation start site). AP2/ERF‐binding GCC box, WRKY‐binding W box, DREB‐binding DRE, and TGA‐binding TGACG motif are indicated. (b) Different‐length *promoter::GUS* constructs (−1975 bp, *2K*); (−3005 bp, *3K*); (−4087 bp, *4K*) used to produce transgenic rice lines and in transient expression analyses. Numbers indicate distance from translation start site. (c) *GUS* mRNA levels in shoots and roots of transgenic rice lines after application of jasmonate (JA, 100 μM). *GUS* mRNA transcript levels relative to those of ubiquitin (*UBQ*) were measured. Experiments were performed three times, each with three seedlings. Asterisk (*) indicates significant difference between JA‐treated plants and JA‐untreated plants (*p *<* *.05; Student's *t* test). (d) Transgenic rice lines harboring *2K*,* 3K*,* 4K‐promoter::GUS* constructs. Two typical lines were obtained for each construct (*2K::GUS*;* 2K‐#7* and *2K‐#11*,* 3K::GUS; 3K‐#14, 3K‐#15*, and *4K::GUS; 4K‐#2, 4K‐#15*). *GUS* mRNA levels in rice roots after JA/salicylic acid (SA) application. Nine‐day‐old rice seedlings were treated with JA (100 μM), JA (100 μM) + SA (100 μM), and SA (100 μM) for 12 h. Experiments were performed three times, each with three seedlings. Asterisk (*) indicates significant difference between JA‐treated plants and JA‐untreated plants (*p *<* *.05; Student's *t* test). Plus symbol (+) indicates significant difference between JA+SA‐treated plants and JA‐treated plants (*p *<* *.05; Student's *t* test). (E) *GUS* mRNA levels in rice roots after application of 1‐aminocyclopropane‐1‐carboxylic acid (ACC)/SA. Nine‐day‐old rice seedlings were treated with ACC (100 μM), ACC (100 μM) +SA (100 μM), and SA (100 μM) for 3 h. Experiments were performed three times, each with three seedlings. Asterisk (*) indicates significant difference between ACC‐treated plants and ACC‐untreated plants (*p *<* *.05; Student's *t* test). Plus symbol (+) indicates significant difference between ACC+SA‐treated plants and ACC‐treated plants (*p *<* *.05; Student's *t* test)

Our preliminary experiments with transgenic rice plants harboring *RSOsPR10 2K‐promoter::GFP* did not show JA/ET‐induced or root‐specific expression of the green fluorescent protein (GFP) reporter. In this study, we searched for known stress‐responsive *cis* elements in the 4‐kb upstream region of *RSOsPR10* using PlantPromoterDB (http://ppdb.agr.gifu-u.ac.jp/ppdb/cgi-bin/index.cgi). We found seven GCC boxes (GCCGCC) [AP2/ERF binding], eight W boxes ((T/C)TGAC(C/T)) [WRKY binding], two TGACG (as‐1‐like) motifs (TGACG) [bZIP type factor (TGA) binding], and four DREs ((A/G)CCGAC) [DREB (AP2/ERF) binding] (Figure 1a). To analyze the promoter activity of the 4‐kb upstream region, we produced transgenic rice lines harboring three different‐length *promoter::*β*‐glucuronidase (GUS)* constructs, *4K* (*−1* to *−4087* *bp::GUS*), *3K* (*−1* to *−3005* *bp::GUS*), and *2K* (*−1* to *−1975 bp::GUS*) (Figure 1b). We checked root‐specific and JA‐inducible *GUS* mRNA expression in the *2K::GUS, 3K::GUS,* and *4K::GUS* transgenic rice lines (Figure 1c). In shoots, no apparent expression of *GUS* transcripts was detected in the *2K::GUS, 3K::GUS,* and *4K::GUS* lines after treatment with and without JA. In roots, very low *GUS* transcript levels were detected in the *2K::GUS* line, even after JA treatment, but *GUS* expression was significantly induced by JA treatment in the *3K::GUS* and *4K::GUS* lines. The JA induction of *GUS* in roots of the *3K::GUS* or *4K::GUS* lines was strongly repressed by SA (Figure 1d). The ethylene biosynthetic precursor 1‐aminocyclopropane‐1‐carboxylic acid (ACC)‐induced *GUS* expression and its suppression by SA were also observed in roots of the *3K::GUS* and *4K::GUS* lines (Figure 1e).

**Figure 2:**
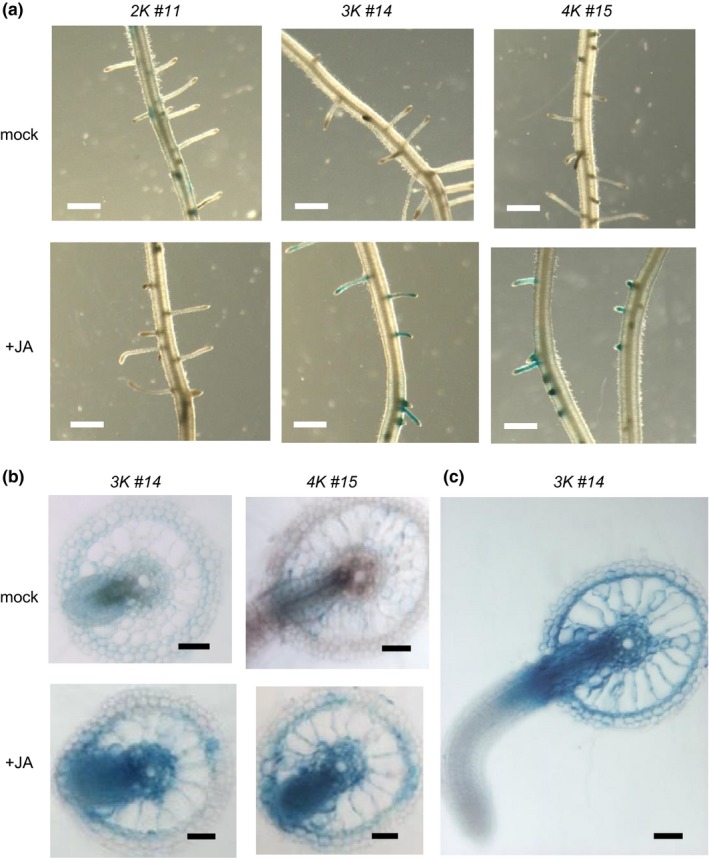
β‐Glucuronidase (GUS) activity in toots of transgenic lines harboring different‐length *RSOsPR10 promoter::GUS* after jasmonate (JA) Treatment. (a) GUS activity in 9‐day‐old seedlings of *2K‐*,* 3K‐,* and *4K‐promoter::GUS* lines treated with or without JA (100 μM) for 12 h. Bars = 50 μm. (b) GUS activity in roots of *3K‐* and *4K‐promoter::GUS* lines (transverse sections of lateral root primordia). Bar = 10 μm. (c) GUS activity in roots of *3K‐* and *4K‐promoter::GUS* lines (transverse sections of growing lateral root). Bar = 10 μm

To determine which promoter regions were required for the tissue‐specific and hormone‐inducible expression of *RSOsPR10* in roots, we conducted histochemical analyses of *promoter::GUS* plants. After JA treatment, GUS activity was observed in lateral roots of the *3K::GUS* and *4K::GUS* lines (Figure 2a), but no GUS activity was detected in the *2K::GUS* plants. In *3K::GUS* and *4K::GUS* plants, intense GUS signals were detected in the lateral root primordia and the basal region of elongating lateral roots (Figure 2b and c). Some GUS signals were also detected in outer and inner endosomal tissues, similar to the patterns observed in a previous immunohistochemical study (Takeuchi et al., 2011).

### 3.2 Deletion analysis of *RSOsPR10* promoter by transient expression in roots using dual LUC assay

**Figure 3:**
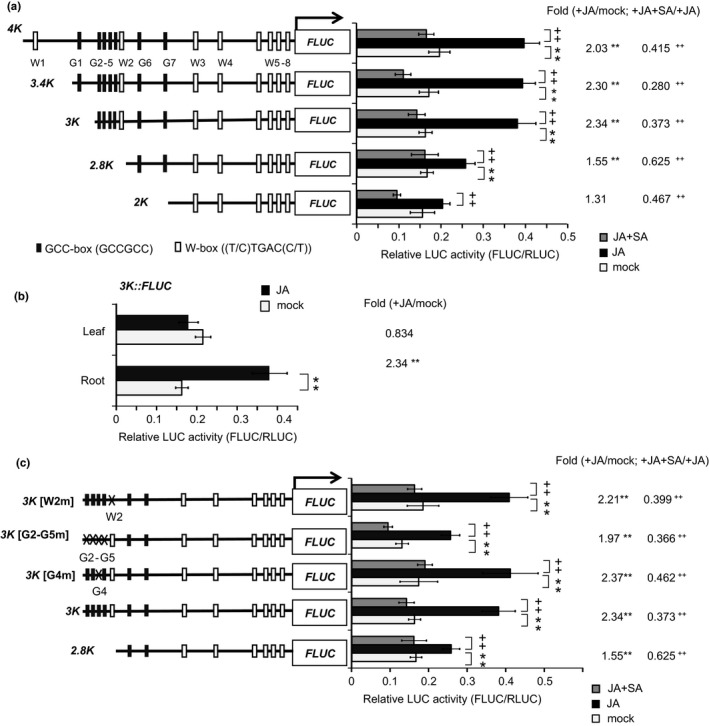
Promoter analysis of *RSOsPR10* using transient dual luciferase (LUC) assay by particle bombardment. (a) *RSOsPR10 2K, 2.8K, 3K, 3.4K, and 4K‐promoter::FLUC* were co‐introduced with *ubiquitin promoter::RLUC* construct into roots of 9‐day‐old rice seedlings by particle bombardment. After 12 h incubation +/− jasmonate (JA, 100 μM) or JA+ salicylic acid (SA, 100 μM), relative LUC activity (FLUC/RLUC) was measured. Black and gray rectangles indicate GCC boxes and W boxes, respectively, in *−*4‐Kb promoter region of *RSOsPR10*. Experiments were performed three times, each with three to six seedlings. Double asterisks (**) indicate significant difference between JA‐treated plants and JA‐untreated plants (*p *<* *.01; Student's *t* test); double plus symbols (++) indicate significant difference between JA+SA‐treated and JA‐treated plants (*p *<* *.01; Student's *t* test). Fold changes are also shown at right side of bars. (b) *RSOsPR10 3K‐promoter::FLUC* and *ubiquitin promoter::RLUC* constructs were co‐introduced into roots or shoots of 9‐day‐old rice seedlings by particle bombardment. After 12‐h incubation with or without JA (100 μM), relative LUC activity was measured. Experiments were performed three times, each with three to six seedlings. Double asterisks (**) indicate significant difference between JA‐treated plants and JA‐untreated plants (**p <* *.01; Student's *t* test). Fold changes are shown at right side of bars. (c) Dual LUC assay of activity of *2.8K‐, 3K‐,* and *3K* promoter sequences with mutations in GCC boxes or W box. *RSOsPR10 promoter::FLUC* constructs and *ubiquitin promoter::RLUC* construct were co‐introduced into roots of 9‐day‐old rice seedlings by particle bombardment. Experiments were performed three to five times, each with three to six seedlings. Double asterisks (**) indicate significant difference between JA‐treated plants and JA‐untreated plants (*p *<* *.01; Student's *t* test). Double plus symbols (++) indicate significant difference between JA+SA‐treated plants and JA‐treated plants (*p *<* *.01; Student's *t* test). Fold changes are shown at right side of bars

To identify important regulatory regions in the *RSOsPR10* promoter, transient expression assays were performed using the *RSOsPR10* promoter linked to the *firefly luciferase* (*FLUC*) reporter. *Renilla LUC* (*RLUC*) controlled by the rice *ubiquitin* promoter was used as an internal control. The ratio of FLUC activity to RLUC activity was calculated as a measure of promoter activity. Various‐length (4‐kb, 3.4‐kb, 3‐kb, 2.8‐kb, and 2‐kb) fragments of the promoter region were fused to *FLUC* (Figure 3a), and these constructs were co‐introduced with *ubiquitin promoter::RLUC* into the roots of 9‐day‐old rice seedlings by particle bombardment. After bombardment, the seedling roots were treated with JA and JA+SA. As shown in Figure 3a, JA inductivity of reporter expression was strong for the *4K*,* 3.4K,* and *3K* promoter constructs, but weak for the *2.8K* promoter construct and almost undetectable for the *2K* construct. For all the *RSOsPR10* promoter constructs, reporter expression was strongly inhibited by SA. In contrast to the roots, shoots showed no JA inductivity of reporter expression with the *3K* promoter construct (Figure 3b). These results indicated that the *3K* promoter region is important for JA induction in the root, consistent with the results obtained using transgenic plants (Figure 1). The −3‐ to −2.8‐kb promoter region (223 bp) (Fig. S1) contains four GCC boxes (G2–G5), recognized by ERF transcription factors in the JA/ET signaling pathway, and one W box (W2), which recognized by WRKY transcription factors in the SA pathway. The ERF factor is thought to function as a transcriptional activator and the WRKY factor as a repressor. Therefore, to examine the roles of the GCC boxes and the W box in this region in JA/ET induction and SA inhibition, respectively, we produced 3‐kb promoter sequences with mutations in the GCC boxes or W box, and fused them with the *FLUC* reporter (Figure 3c). The promoters with single mutations, *3K*[G4 m] or *3K*[W2 m], resulted in reporter expression levels similar to those obtained with the control *3K* promoter, with JA‐induced expression levels almost double those in the mock‐treated plants. Fold changes of +JA/mock with *3K*,* 3K*[G4 m], and *3K*[W2 m] were 2.34, 2.37, and 2.21, respectively. However, the expression level of *3K*[G2–G5 m] after JA treatment (fold change, 1.97) was lower as that of *2.8K* promoter (fold change, 1.55) (Figure 3c).” These results showed that the four GCC boxes are important for JA/ET inductivity of *RSOsPR10*. The mutation in W2 had no inhibitory effect, indicating that this W box is not involved in suppressor activity. Instead, we hypothesized that the other six W boxes within the 2‐kb upstream from the translational start site might be responsive to SA repression. The SA repression observed in plants harboring the *2K* promoter sequence (Figure 3a) supported this hypothesis.

### 3.3 Effector assays to determine effects of OsERFs on *3K::LUC* expression

**Figure 4:**
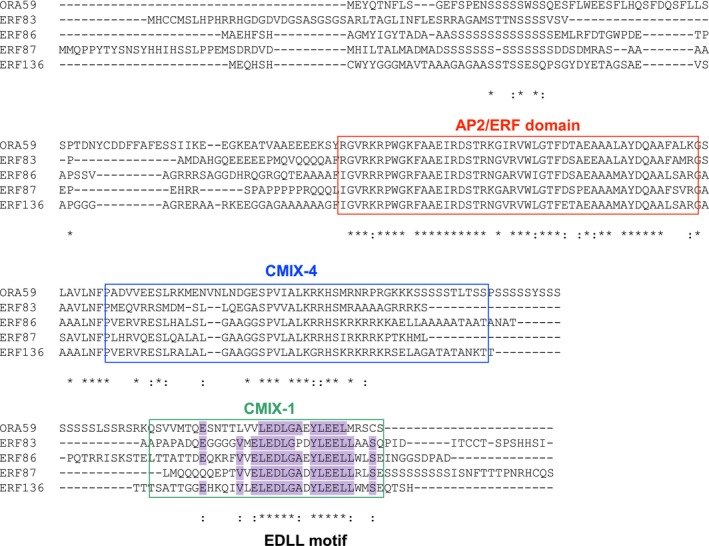
Alignment of amino acid sequences of arabidopsis ORA59 and its orthologs in rice, OsERF83, OsERF86, OsERF87, and OsERF136, in group IXc ERF superfamily. Sequences were obtained and aligned using Clustal Omega (http://www.ebi.ac.uk/Tools/msa/clustalo/). Common domains are framed with red (AP2/ERF domain), blue (CMIX‐4), and green (CMIX‐1). EDLL motif is marked in CMIX‐1 domain (purple; residues are conserved in four (:) or five (*) sequences)

**Figure 5:**
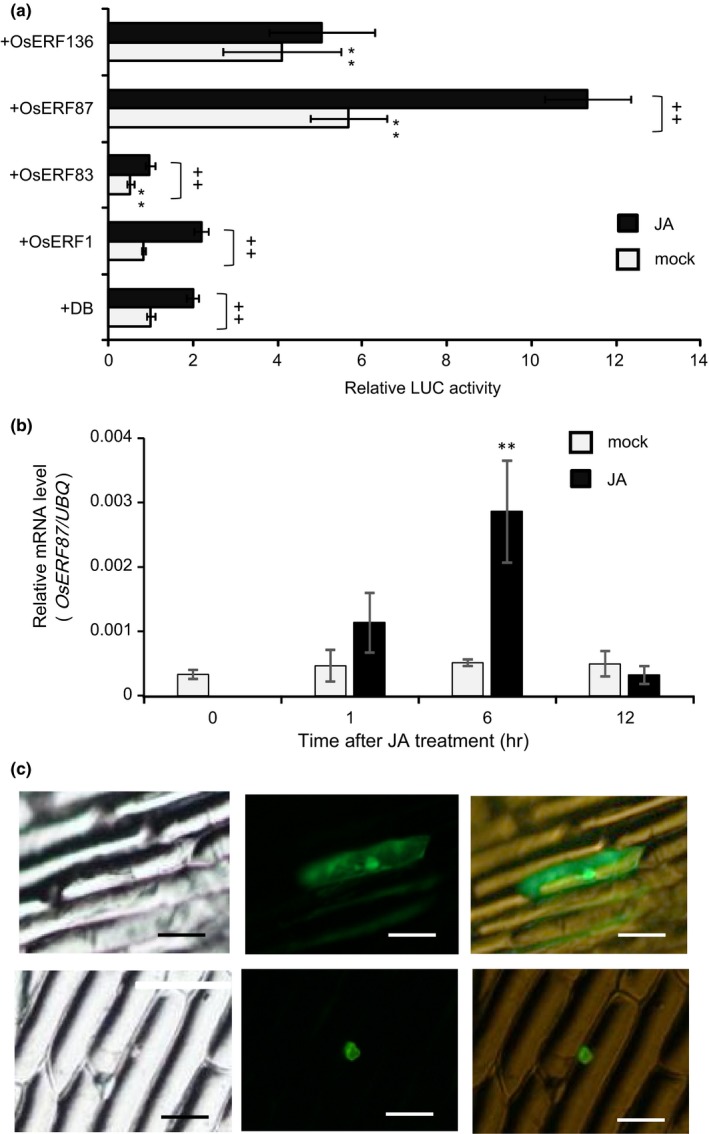
Effect of OsERFs on transcriptional activity of *RSOsPR10 3K* promoter. (a) For dual LUC assay, *RSOsPR10 3K‐promoter::FLUC* and *ubiquitin promoter::RLUC* were used. DNA‐binding domain of GAL (DB) was used as control, and *OsERF1, OsERF83, OsERF87,* and *OsERF136* constructs were used as effectors. *OsERFs* were driven by *CaMV 35S* promoter. Each combination of constructs was introduced into rice roots, and the seedlings were incubated for 12 h with or without jasmonate (JA). Ratios of FLUC to RLUC were measured and are shown relative to that in DB. Experiments were performed three times, each with three to five seedlings. Double asterisks (**) indicate significant difference between *OsERF1, OsERF83, OsERF87,* or *OsERF136* construct and *DB* control (*p *<* *.01; Student's *t* test). Double plus symbols (++) indicate significant difference between JA‐treated roots and JA‐untreated roots (*p *<* *.01; Student's *t* test). (b) *OsERF87* expression in roots after JA treatment. Experiments were performed with four seedlings. Asterisks indicate significant difference between JA‐untreated plants and JA‐treated plants (**p <* *.01; Student's *t* test). (c) Nuclear localization of GFP‐fused OsERF87 protein in onion epidermal cells. Left, visible light field; middle, green fluorescence field; right, merged. Upper panels, GFP control; lower panels, OsERF87‐GFP. Bar = 200 μm

Transactivation assays were performed to investigate which transcription factor(s) are involved in regulating *RSOsPR10*. In our previous study, we proposed that OsERF1 induces *RSOsPR10* expression by binding to GCC box(es) in the promoter (Takeuchi et al., 2011). In this study, OsERF1 did not promote the expressional activity of the 3‐kb *RSOsPR10* promoter construct (as shown later in Figure 5a). In *Arabidopsis*, an ERF factor, ORA59, was shown to control the expression of the JA‐inducible marker gene *PDF1.2* through directly binding to GCC boxes in its promoter (Huang, Catinot, et al., 2016; McGrath et al., 2005; Van der Does et al., 2013; Zander et al., 2014; Zarei et al., 2011). We searched the ERF proteins to identify ORA59 orthologs in rice and focused on four ERFs in group IXc (OsERF83, OsERF86, OsERF87, and OsERF136). Comparisons of protein structures between ORA59 and these four OsERFs (Figs 4 and S2) revealed specific CMIX‐4 and CMIX‐1 domains in the rice ERFs as well as a common AP2/ERF domain [SALAD (http://salad.dna.affrc.go.jp/salad/)]. *OsERF83*,* OsERF87,* and *OsERF136* mRNA transcripts were relatively abundant in roots and exhibited clear JA inductivity in roots [RiceXpro database (http://ricexpro.dna.affrc.go.jp/)] (Fig. S3).

We cloned *OsERF1*,* OsERF83*,* OsERF87,* and *OsERF136* and investigated the effect of these OsERFs on 3‐kb promoter activity by transient expression assays, co‐introducing the *ERF* genes as effectors into roots of rice seedlings harboring *3K::FLUC* (Figure 5a). Transactivation activity of OsERF1 was not detected, regardless of JA treatment, compared with the control (DNA‐binding domain of GAL (DB)). OsERF83 showed significant transrepression activity with or without JA treatment. Conversely, both OsERF87 and OsERF136 showed clear transactivation activity without JA treatment, but OsERF87 only showed enhanced expression of the 3‐kb promoter under JA treatment. Induction of *OsERF87* mRNA expression in JA‐treated rice roots was confirmed (Figure 5b), and nuclear localization of OsERF87‐GFP was observed in onion epidermal cells (Figure 5c). These results strongly suggested that OsERF87 and OsERF136 act as positive regulators of *OsRSPR10* expression, and OsERF87 is a most likely candidate for a JA/ET‐dependent transcriptional activator of *RSOsPR10*, presumably via binding to GCC box(es) in its 3‐kb promoter region.

### 3.4 Repression of *3K::LUC* by OsWRKYs

**Figure 6:**
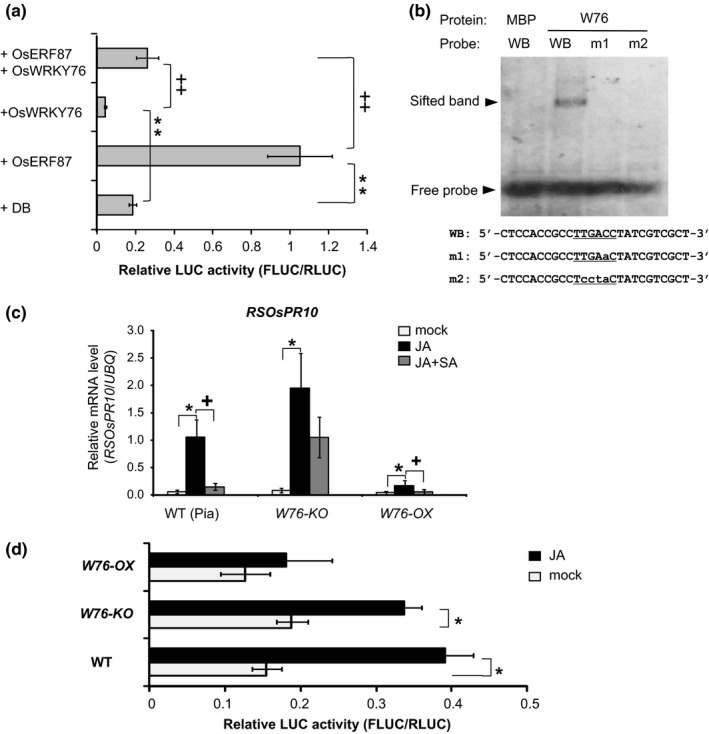
Effect of OsWRKY76 on transcriptional activity of *RSOsPR10 3K* promoter. (a) For dual LUC assay, *RSOsPR10 3K‐promoter::FLUC* and *ubiquitin promoter::RLUC* and effector constructs OsWRKY76 and OsERF87 (GAL4 DB as control) were co‐introduced as shown in Figure 3. Experiments were performed three times, each with three to six seedlings. Asterisks (**) indicate significant difference between GAL4 DB control and OsERF87 or OsWRKY76 as effector (*p *<* *.01; Student's *t* test); plus symbols (++) indicate significant differences in reporter expression levels between OsERF87 or OsWRKY76 and OsERF87 + OsWRKY76 as effectors (*p *<* *.01; Student's *t* test). (b) Gel mobility shift assay of recombinant OsWRKY76 protein fused to MBP (W76) and MBP control. DIG‐labeled W‐box core sequence in −3‐ to −2.8‐kb promoter region (WB) and mutated W‐box core sequences (m1 and m2). (c) *RSOsPR10* expression in *OsWRKY* mutants. Expression levels of *RSOsPR10* relative to ubiquitin (*UBQ*) in *OsWRKY76*‐knockout (*W76‐KO*) and *OsWRKY76*‐overexpressing (*W76‐OX*) rice mutants were measured by qRT‐PCR. mRNA was extracted from roots of 9‐day‐old rice seedlings treated with jasmonate (JA, 100 μM), JA (100 μM) + salicylic acid (SA, 100 μM), or SA (100 μM) for 12 h. Experiments were performed three times, each with three plants. Asterisk (*) and plus symbol (+) indicate significant differences between JA‐treated plants and JA‐untreated plants and between JA+SA‐treated plants and JA+SA‐untreated plants, respectively (*p *<* *.05; Student's *t* test). (d) Effect of JA treatment on *RSOsPR10 3K::FLUC* expression in *WRKY76‐KO* and *WRKY76‐OX* rice roots. Experiments were performed three times, each with three to eight seedlings. Asterisk (*) indicates significant difference between JA‐treated roots and JA‐untreated roots (*p *<* *.05; Student's *t* test)

Next, we tried to identify the factor(s) responsible for the SA suppression of JA/ET‐induced *RSOsPR10* transcription. Based on the results of our previous work, we presumed that SA induced some OsTGA or OsWRKY proteins to repress JA/ET‐dependent *RSOsPR10* expression (Takeuchi et al., 2011). In fact, WRKY factors are known to function not only as positive regulators but also as negative regulators by targeting a number of defense‐related genes and regulating their expression through binding to W box(es) in their promoters (Birkenbihl, Kracher, & Somssich, 2017; Liu, Chen, et al., 2016). As shown in Figures 1 and 3, the 3‐kb promoter region closely mimicked the in vivo expression pattern of *RSOsPR10,* that is, JA/ET induction, SA suppression, and root‐specific expression. There are seven W‐box motifs within the 3‐kb *RSOsPR10* promoter region. Thus, we hypothesized that some OsWRKY(s) may directly inhibit *RSOsPR10* expression at the transcriptional level through binding to the W box(es) in its promoter. *OsWRKY76* in rice was reported to exhibit transcriptional repressor activity in a W‐box‐dependent manner, and to suppress the induction of several *PR* genes, such as *OsPR1*,* OsPR10b,* and *OsPR15*, which have W boxes in their promoter regions (Yokotani et al., 2013). Therefore, in this study, we examined the effect of OsWRKY76 on 3‐kb *RSOsPR10* promoter activity using dual effector assays. OsERF87 significantly induced the expressional activity of the 3‐kb promoter, and co‐expression of OsWRKY76 strongly inhibited this induction (Figure 6a). Moreover, the expression level with OsWRKY76 was lower than that in the DB control without SA treatment, suggesting that OsWRKY76 represses the initiation event of *RSOsPR10* transcription. These results indicated that OsERF87 in the JA/ET pathway acts as a transcriptional activator for *RSOsPR10* induction, while OsWRKY76 in the SA pathway functions as a repressor of *RSOsPR10* transcription.

To analyze sequence‐specific DNA‐binding activity in vitro, OsWRKY76 was prepared as a fusion protein with maltose‐binding protein (MBP) in *Escherichia coli* and its binding activity was tested in a gel mobility shift assay. As shown in Figure 6b, the MBP‐fused OsWRKY76 protein bound to the digoxigenin (DIG)‐labeled W‐box core sequence in the −3‐ to −2.8‐kb promoter region (WB), but MBP control did not. Furthermore, MBP‐OsWRKY76 did not bind to the probes with a mutated W‐box (m1 and m2). These results demonstrated that OsWRKY76 can directly and specifically bind to the W box in the *RSOsPR10* promoter, as previously reported showing its binding to synthetic W‐box sequence (Yokotani et al., 2013). However, the mutation in W2 at around 3 kb in the promoter did not affect gene expression levels (Figure 3c, indicating that other W boxes (W3–W8) may function to suppress promoter activity.

Subsequently, we checked the expression level of *RSOsPR10* in the roots of *OsWRKY76*‐knockout (*KO*) and *OsWRKY76*‐overexpressing (*OX*) rice lines using qRT‐PCR. The *OsWRKY76* transcript levels in wild‐type plants were very low in mock and JA treatment, but apparently induced by SA, while the levels were about 10 times higher in *OsWRKY76‐OX* plants than in wild type, even mock and JA treatment (Fig. S4). No JA inductivity of *OsWRKY76* in roots of rice seedlings is also shown [RiceXpro database (http://ricexpro.dna.affrc.go.jp/)] (Fig. S5). In the *OsWRKY76‐OX* plants, *RSOsPR10* expression was strongly inhibited, even in JA‐treated plants (Figure 6c). By contrast, *RSOsPR10* expression under JA treatment was moderately upregulated and the repression by SA was apparently reduced in the *OsWRKY*76*‐KO* plants (Figure 6c). Promoter analyses were also conducted by transiently expressing the dual LUC reporter in the roots of *OsWRKY76*‐*OX* or *OsWRKY76*‐*KO* rice seedlings. The levels of FLUC expression after JA treatment were significantly increased in wild‐type and *OsWRKY76‐KO* plants, but the level did not change in *OsWRKY76‐OX* plants (Figure 6d). These results indicated that OsWRKY76 is a repressor of *RSOsPR10* transcription in vivo.

## 4 DISCUSSION

### 4.1 3‐kb promoter region is responsible for JA/ET induction, SA suppression, and lateral root‐specific expression of *RSOsPR10*

Worldwide, biotic and abiotic stresses seriously affect the productivity of important crops such as rice. Prior or simultaneous exposure to one type of stress can affect plants’ responses to other stresses, indicating that there is extensive overlap and crosstalk among stress response signaling pathways. Many studies have focused on the crosstalk between JA/ET and SA signaling to control the expression of pathogenesis‐related (PR) proteins in the model plant *Arabidopsis* (Thaler et al., 2012). However, far fewer studies have focused on the antagonistic regulation of *OsPR* expression by JA/ET and SA in rice. Our research has provided clear evidence that *RSOsPR10* is induced by the JA/ET pathway and that SA strongly inhibits its induction in roots (Hashimoto et al., 2004; Takeuchi et al., 2011). In a previous study, treatments with 100 mM NaCl, drought stress, and JA induced the accumulation of RSOsPR10 specifically in root cortex tissues, while there was no expression in nontreated control roots (Takeuchi et al., 2011). In addition, *RSOsPR10*‐antisense and *RSOsPR10‐KO* were lethal for rice, while *RSOsPR10‐OX* rice showed increased root number, greater root mass, and increased drought tolerance (Takeuchi et al., 2016). The results of the present study also showed that the promoter region, at least 3 kb, of *RSOsPR10* drives root‐specific gene expression in lateral root primordia and at the basal region of growing lateral roots (Figure 6). Several other studies have suggested that JA and auxin signaling and their crosstalk are important in the formation and growth of lateral roots (Ito et al., 2016; Lu et al., 2015; Sun et al., 2009). Therefore, we hypothesize that RSOsPR10 is one of the key molecules in root development. Further studies on the physiological roles and molecular functions of *RSOsPR10* could reveal mechanisms of root development and the formation of root system architecture, leading to the engineering of stress‐tolerant rice.

In the rice genome, there are five *OsPR10* homologs (*RSOsPR10, PBZ1‐like OsPR10, OsPR10b, OsPR10,* and *OsPR10a/PBZ1*) located in a gene cluster in chromosome 12 (Fig. S6). According to the RiceXpro database (http://ricexpro.dna.affrc.go.jp/), these five genes are all induced by pathogen infection, but their expressions are differently regulated by plant hormones and in different organs. For example, *OsPR10a/PBZ1* and *OsPR10* are upregulated by both SA and JA, and expressed abundantly in aerial parts. In contrast, the *OsPR10b* expression pattern is very similar to that of *RSOsPR10*, but its expression level is about 1/40 that of *RSOsPR10* (Fig. S7). Analyses of the promoter regions revealed that the *RSOsPR10* and *OsPR10b* promoters have a GCC box at around −3 kb and several W boxes within 1–2 kb of the transcription initiation site (Fig. S6). Although there have been no detailed studies on the role of this gene cluster and the molecular and in vivo functions of their encoded genes, we speculate that these PR10 proteins may function redundantly in biotic and abiotic stress responses.

### 4.2 OsERF87, an ortholog of *Arabidopsis* ORA59, activates *RSOsPR10* transcription by binding to GCC Box(es) in 3‐kb promoter region

**Figure 7:**
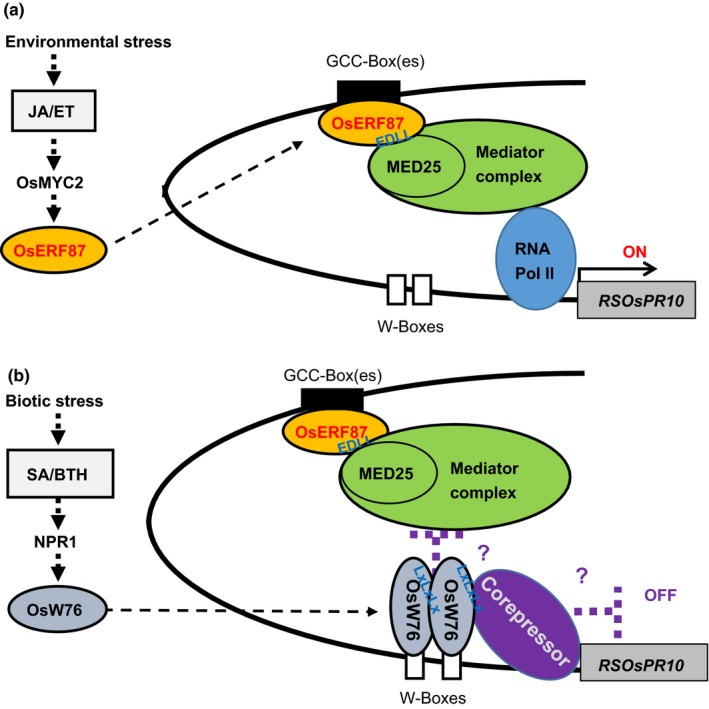
Proposed model for transcriptional regulation of *RSOsPR10* via jasmonate/ethylene (JA/ET) and salicylic acid (SA) pathways in rice roots. (a) In rice seedlings exposed to environmental stress (drought, salinity, or wounding), JA‐induced *OsERF87* expression in roots increases and OsERF87 binds to GCC boxes in −3‐ to −2.8‐kb region of *RSOsPR10* promoter (broken arrows). OsERF87 interacts with mediator complex by binding to MED25 via EDLL motif and activates RNA polymerase II. Finally, RSOsPR10 levels increase in the root and the seedling gains higher tolerance against moderate and long‐term stresses. (b) Under such stress conditions, if the seedling is also under pathogen attack, the increased SA/BTH level induces *OsWRKY76* expression (broken arrows). OsWRKY76 as a homodimer binds to W boxes in the 0 to −1‐kb region of the *RSOsPR10* promoter. OsWRKY76 recruits a “corepressor” via the EAR‐like motif (LxLxLx) in the N‐terminus of OsWRKY76. The corepressor directly inhibits RNA polymerase or the mediator complex to stop *RSOsPR10* transcription, as indicated (thick broken lines and bars). By this process, seedlings can allocate more energy to pathogen defense

Based on the results of genomewide analyses of the large ERF families in *Arabidopsis* and rice, their members can be divided into 12 (I–Xb; in *Arabidopsis*) and 15 (I–XIV; in rice) groups (Nakano, Suzuki, Fujimura, & Shinshi, 2006; Rashid, Guangyuan, Guangxiao, Hussain, & Xu, 2012). Among these, the group IXc ERF members are known to promote the immune response in *Arabidopsis*. Five Arabidopsis ERFs (ORF59, ERF1, AtERF14, AtERF15, and TDR1) in group IXc were shown to activate transcription by binding to a component of the mediator complex (the eukaryotic transcriptional activator complex) (Cevik et al., 2012; Huang, Catinot, et al., 2016; Kazan, 2017). The mediator complex functions as a bridge between DNA‐binding activators and the RNA polymerase II complex, and MEDIATOR25 (MED25) acts as an integrative hub for the regulation of JA/ET‐responsive gene expression. Previously, the binding between ERFs and the MED25 was shown to be mediated by the EDLL motif in the CMIX‐1 domain, specific to group IXc ERF members (Huang, Catinot, et al., 2016; Tiwari et al., 2012). Although many studies have focused on the roles of OsERFs in various biotic and abiotic stress responses of rice, none has been studied on group IXc ERFs (Rashid et al., 2012). In the present study, we found a pivotal role of OsERF87, a group IXc ERF, in JA/ET‐induced *RSOsPR10* expression. This is the first report of the involvement of OsERF87 as a transcriptional activator in rice. The results showed that OsERF87 functions in JA/ET induction of *RSOsPR10*, presumably through binding to GCC box(es) in the −3‐ to −2.8‐kb promoter region (Figures 3 and 5). Arabidopsis ORA59 and all the rice group IXc ERFs (OsERF83, OsERF86, OsERF87, and OsERF136) also contain the EDLL motif in their CMIX‐1 domain (Figure 4). These facts indicate that rice group IXc ERFs may function as transcriptional activators by binding to the mediator complex via the EDLL and MED25 interaction, as illustrated in our proposed model (Figure 7).

In *Arabidopsis*, MED25 is also involved in the regulation of gene expression by several plant hormones; it binds to ARFs in the auxin signaling pathway, ABI5 and DREB2A in the ABA signaling pathway, and MYCs in the JA signaling pathway (Cevik et al., 2012; Ito et al., 2016; Kazan, 2017). To date, almost all studies on the function of MED25 have been restricted to *Arabidopsis* and wheat (Fitzgerald et al., 2015; Liu, Zhang, Jia, & Sun, 2016). Although there have been no reports on rice MED25, in silico data show that rice *MED25* is ubiquitously expressed throughout rice plants (RiceXpro database (http://ricexpro.dna.affrc.go.jp/)) (Fig. S8). Its important roles and diverse and multiple functions in rice will be soon clarified.

### 4.3 OsWRKY76 functions as a transcriptional repressor of *RSOsPR10* expression

The members of the plant‐specific WRKY transcription factor family contain one or two highly conserved WRKY amino acid sequences at the N‐terminus and a zinc finger motif in the C‐terminal region (Eulgem, Rushton, Robatzek, & Somssich, 2000; Ross, Liu, & Shen, 2007; Rushton, Somssich, Ringler, & Shen, 2010). Most WRKYs bind to a consensus *cis* element known as the W box ((T/C)TGAC(C/T)) and mainly act as positive transcription factors, although group IIa WRKYs exhibit negative functions. In rice, the WRKY IIa subfamily has four members (OsWRKY28, OsWRKY62, OsWRKY71, and OsWRKY76), at least three of which (OsWRKY28, OsWRKY62, and OsWRKY76) function as transcriptional repressors in blast disease resistance (Chujo et al., 2013; Fukushima, Mori, Sugano, & Takatsuji, 2016; Liu, Chen, et al., 2016; Liu, Zhang, et al., 2016; Peng, Bartley, Canlas, & Ronald, 2010; Yokotani et al., 2013). The gene expression of these *OsWRKYs is* known to be upregulated by blast fungus inoculation and benzothiadiazole (BTH) treatment, and therefore, it is probable that the regulation is downstream of the SA signaling pathway. However, it was also reported that *OsWRKY76* was induced by wounding and MeJA in rice seedlings, indicating the involvement of JA signaling pathway (Liu, Chen, et al., 2016; Liu, Zhang, et al., 2016; Yokotani et al., 2013). These results were obtained with leaves and shoots, but studies with roots have not been performed so far. In the present study, we checked the effect of JA and SA treatments on *OsWRKY76* expression in roots of wild‐type seedlings and the result showed that it was induced by SA treatment but not by JA treatment (Fig. S4). No response against JA on *OsWRKY76* induction in rice roots was also shown in RiceXpro database (Fig. S5). Therefore, it is likely that *OsWRKY76* induction by wounding and/or JA is differently regulated in shoots and roots. The nuclear localization of these OsWRKYs and their transcriptional repression by binding to W box(es) were previously observed in rice leaves (Chujo et al., 2013; Yokotani et al., 2013). In the present study, SA‐inducible OsWRKY76 significantly suppressed *3K RSOsPR10* promoter activity and strongly inhibited OsERF87 induction of gene expression in roots (Figure 6a). Moreover, the expression of *RSOsPR10* was strongly suppressed in *OsWRKY76‐OX* rice, but not significantly suppressed by SA in *OsWRKY76‐KO* lines, confirming the in vivo function of OsWRKY76 in rice roots (Figure 6c). The repressor function of OsWRKY28 and OsWRKY76 was predicted by the presence of the LxLxLx putative repressor motif in the N‐terminus (Chujo et al., 2013) (Fig. S9). The LxLxLx motif was reported to associate with the amphiphilic repression (EAR) domain found in almost all AUX/IAA members and some ERFs (Li, Tiwari, Hagen, & Guilfoyle, 2011; Ohta, Matsui, Hiratsu, Shinshi, & Ohme‐Takagi, 2001; Tiwari, Hagen, & Guilfoyle, 2004) (Table S1). The AUX/IAAs bind to a corepressor, TPL, to inactivate ARF function (Ito et al., 2016); however, there have been no reports on how this binding to the corepressor is accomplished. More recent results of a study on alternative splicing variants of *OsWRKY62* and *OsWRKY76* transcripts indicated that short variant of OsWRKY76.2 with the LxLxLx motif at the N‐terminus exhibited repressor activity, but OsWRKY62 lacking the LxLxLx motif at the N‐terminal also had repressor activity, which was attributed to two short sequences, PTDDS and EDLEEK (Fig. S9) (Liu, Chen, et al., 2016). Further research is required to identify the amino acid sequence motif(s) responsible for the repressor activity of these OsWRKYs and to determine which protein is the corepressor.

### 4.4 A model for transcriptional regulation of *RSOsPR10* by two DNA‐binding factors, OsERF87 and OsWRKY76 in rice roots

There is a huge number of transcription factors in eukaryotes, but most have been studied one by one or in clusters of a few related factors (Song et al., 2016; Weirauch et al., 2014). The importance of the cooperative binding of transcription factors has been recognized in developmental processes and environmental adaptation of multicellular organisms. However, it has not been studied in detail, especially at the three‐dimensional structural level (Morgunova & Taipale, 2017). As it was discovered that the lengths of promoter regions are longer than a few kb from the transcription start sites in many eukaryotic genes, researchers have argued that there must be protein complexes with multiple transcription factors and related proteins in the loop structure of double‐stranded DNAs. This is important aspect of understanding how gene expression is precisely regulated according to developmental processes and various environmental changes. In this study, we focused on the activity of two transcription factors, the OsERF87 activator that may bind to GCC box(es) in the −3‐ to −2.8‐kb region of *RSOsPR10* promoter, and the OsWRKY76 repressor that binds to W box(es) within the 2‐kb region upstream of the translation start site (Figures 1 and 3). As discussed above, OsERF87 could interact with relatively large mediator complexes to activate RNA polymerase II for transcription of *RSOsPR10*. If this is the case, there is enough space to permit OsWRKY76 binding to the promoter in a region closer to the translation start site. As reported previously, *Arabidopsis* and rice WRKY proteins in groups II and III often form homo‐ and heterodimers and the forms are suggested to affect their transcriptional activity (Chi et al., 2013; Liu, Chen, et al., 2016; Liu, Zhang, et al., 2016). The OsWRKY dimer may recruit a corepressor that represses directly *RSOsPR10* transcription, or may inhibit the OsERF87‐mediator complex activity (Figure 7). In *Arabidopsis*, ORA59 binds to the GCC box around 300‐bp upstream of the transcription initiation site of *PDF1.2* (Zander et al., 2014). Because there is no W box in the *PDF1.2* promoter, it is likely that some repressor factor(s) other than WRKY(s) inhibits *ORA59* expression or the ORA59‐mediator complex (Kazan, 2017). In the case of regulation by the ARF7/19‐AUX/IAA14‐mediator complex, ARFs bound to the promoter at around 700‐bp upstream of the auxin‐responsive target gene (Ito et al., 2016). Compared with that case, OsERF87 binding at 2.8‐kb upstream of *RSOsPR10* leaves enough space by making a loop structure. Because the 3‐kb promoter region contains many other *cis* elements, such as G‐box‐like, DRE, TGACG, and W‐box like motifs, it is possible that a complex combination of protein factors regulates the expression of *RSOsPR10* according to developmental and/or environmental dynamic changes.

The results of this study show that *RSOsPR10* expression is regulated antagonistically by the novel OsERF87 activator and OsWRKY76 repressor in JA/ET and SA pathways, respectively. We propose the following model for the mechanism of action (Figure 7): When rice plants are subjected to environmental stresses, such as drought, high salt, and wounding, JA/ET‐inducible OsERF87 induces *RSOsPR10* expression in the root, and RSOsPR10 enhances lateral root growth and development. The increased root mass leads to higher tolerance against biotic stresses. However, when rice plants are infected by pathogens, the SA level increases throughout the plant. The increased SA results in rapid induction of OsWRKY76 in the root, strongly suppressing the expression of *RSOsPR10*. By this process, rice plants can allocate more energy to defense against pathogen infection. This mechanism of antagonistic regulation is compatible with the concept a cost balance, because defenses are costly to produce (Ahmad et al., 2016; De Vleesschauwer et al., 2013; Thaler et al., 2012; Vos et al., 2015).

## AUTHOR CONTRIBUTIONS

T.K. conceived and planned the project. T.K., K.O., K.M., H.Y., and Y.N. designed the research. T.Y., A.G., K.N., M.T., Y.Y., H.S., T.O., T.N., N.Y., E.M., and K.M. performed the experiments. T.K., T.Y., A.G., H.S., K.M., and Y.N. wrote the article with contributions from all the authors.

## Supporting information


** **
