# Supplementary material for: Expression of RSOsPR10 in rice roots is antagonistically regulated by jasmonate/ethylene and salicylic acid via the activator OsERF87 and the repressor OsWRKY76, respectively
Source: Plant Direct. 2018 Mar 30;2(3):e00049. doi: 10.1002/pld3.49 (PMC6508531; doi:10.1002/pld3.49)
Supplement: Supplementary file 1 [file PLD3-2-e00049-s001.pdf]

Supplemental Figure 1

```
     -3050      -3040      -3030      -3020      -3010      -3000
CTCTCTCTCT CCATTCTCGA TCCCTCCTCC CACCTCTCCC AGCTCTGGGT GGTGGTGCTC
                                                           3K

     -2990      -2980      -2970      -2960      -2950      -2940
CAGCATGCAA CGGTGCCCAG CGGCGGCACA CCTAGTAGCC GGATCGAGGC GGCGGTGCGC
                        G2                          G3

     -2930      -2920      -2910      -2900      -2890      -2880
AGGCGGATCG AGGCGGCTGC CATGCTCGGC GGCGGTTAGA TCAGAGGCGA GGGCTCGCAG
               G4                G5

     -2870      -2860      -2850      -2840      -2830      -2820
CGACGATAGG TCAAGGCGGT GGAGTGCGGA TCCAAGGCCA CGCGAGCTCT GCCATCCACA
            W2

     -2810      -2800      -2790      -2780      -2770      -2760
ACGACCTCGA CCTACATCCA CATCCGGCAG CGGCTTCAGC TCCACCCCAT CCCTATGCCC
                                     2.8K
```

Nucleotide sequence of the region from −3 kb to −2.8 kb (223 bp) upstream of the ATG translation initiation site of *RSOsPR10*.

The four GCC-boxes and one W-box are marked by underlines.

Supplemental Figure 2

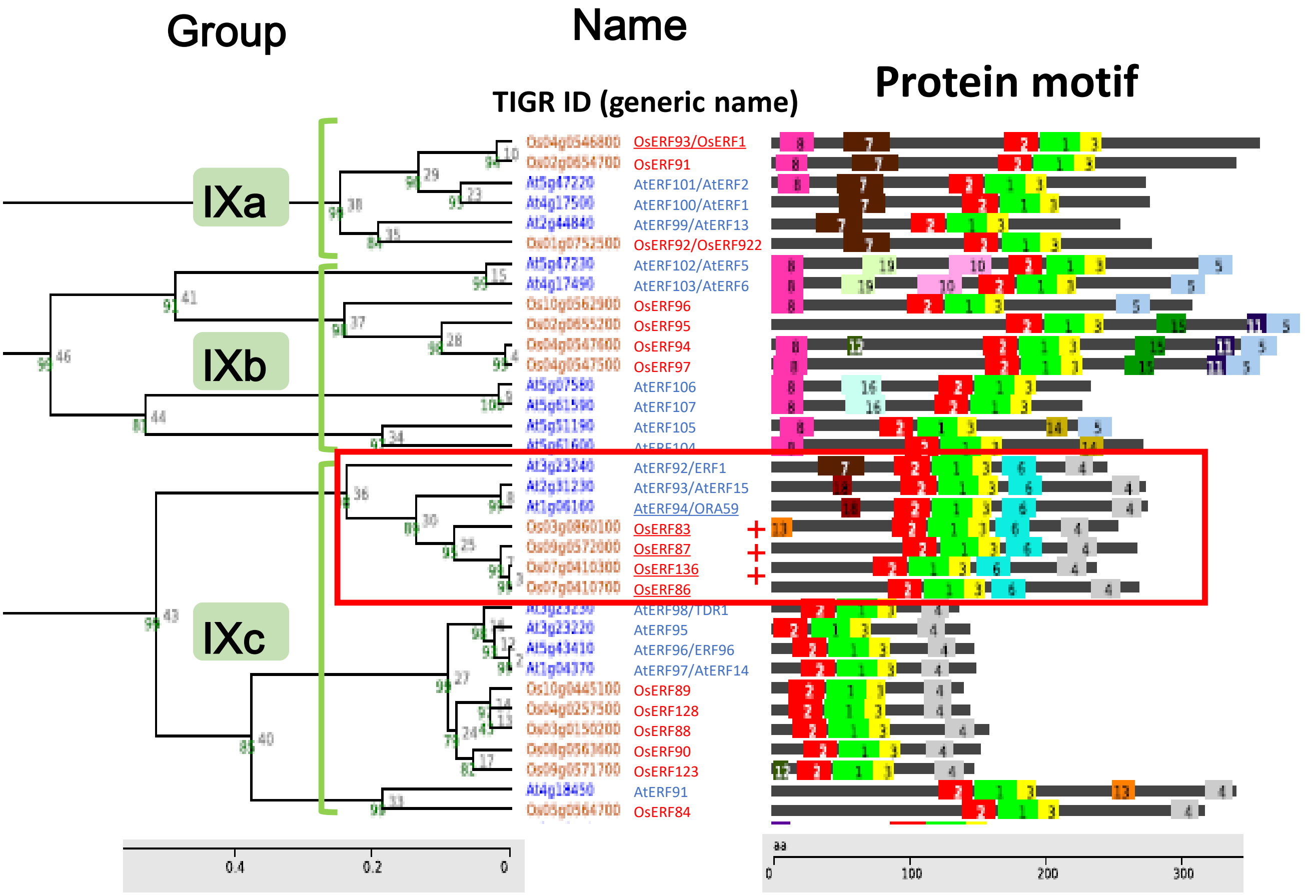

Phylogenic tree and protein motif of ERF family group IX in *Arabidopsis* (blue) and rice (red).

Arabidopsis ORA59/AtERF94 is reported it's involvement on JA/ET-SA antagonistic regulation of PDF.1.2 (PR protein) (Pre' et al., 2008 ; Zarei et al., 2011 ; Zander et al.). Rice orthologs (OsERF83, 87, 136, 86) of Arabidopsis ORA59 have similar protein motifs, containing a specific motif 6 (blue) and longer amino acid sequences than lower subgroup of IXc . Generic name of ERFs is referred by Nakano et al., 2006 and root specific JA inductivity reported in RiceXPro (http://ricexpro.dna.affrc.go.jp/category-select.php) are indicated as "+" (after 100 µM JA treatment within 12 h).

Supplemental Figure 3

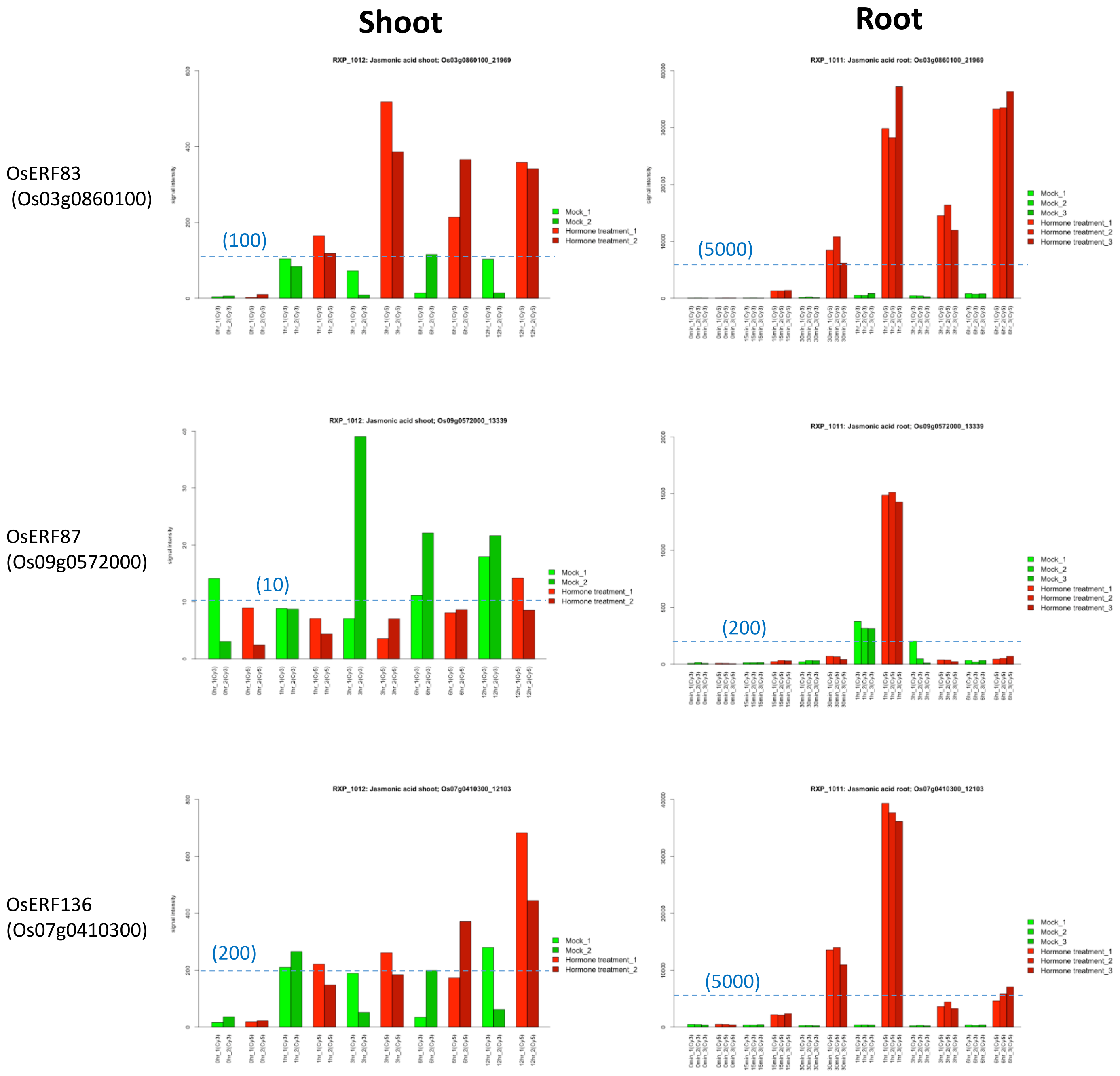

Expression profiles of *OsERF* group IXc genes, *OsERF83, OsEFR87, OsERF136,* in shoots and roots after JA treatment.
Seven-day-old rice (*Oryza sativa* L. japonica cv. Nipponbare) seedlings were treated with 100 µM jasmonate and RNA expression was observed for 12 hr after jasmonate treatment. The data were obtained from RiceXPro (http://ricexpro.dna.affrc.go.jp/category-select.php).

Supplemental Figure 4

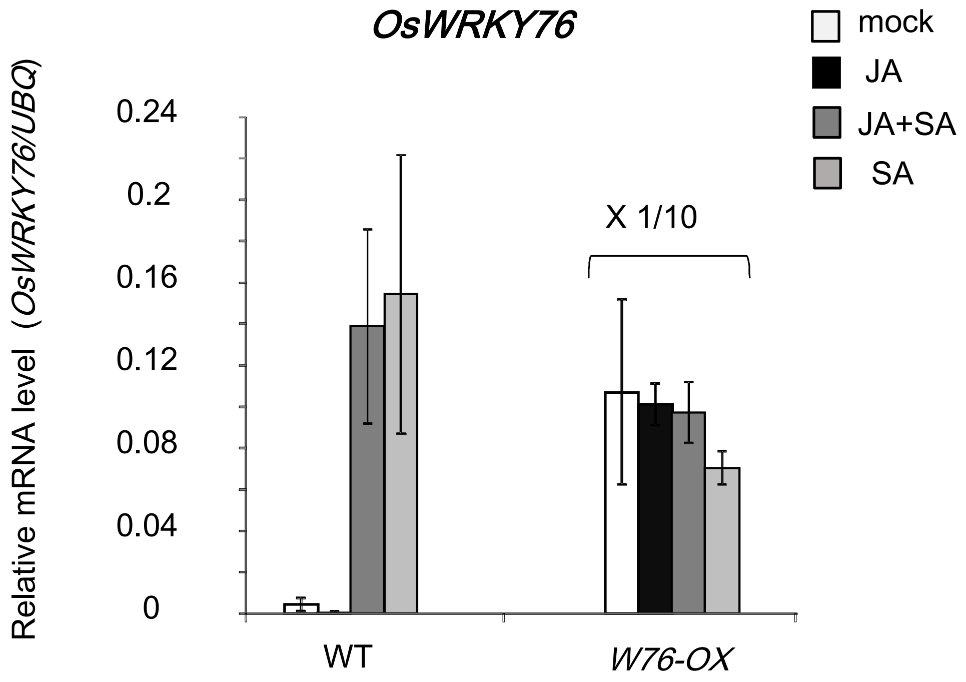

***OsWRKY76* gene expression in the root of wild-type and *OsWRKY76-OX* plants.**

In wild-type (WT) and *OsWRKY76-OX (W76-OX)* rice (Pia background), *OsWRKY76* mRNA expression levels relative to ubiquitin (UBQ) were measured by qRT-PCR. mRNA was extracted from the roots of 9-day-old rice seedlings treated with JA (100 µM), JA (100 µM) + SA (100 µM), and SA (100 µM) for 12 h.

Supplemental Figure 5

Shoot

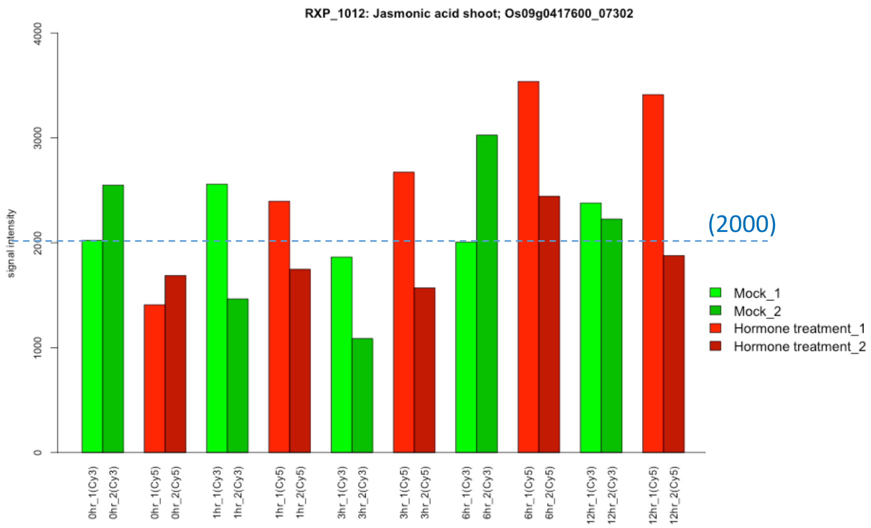

Root

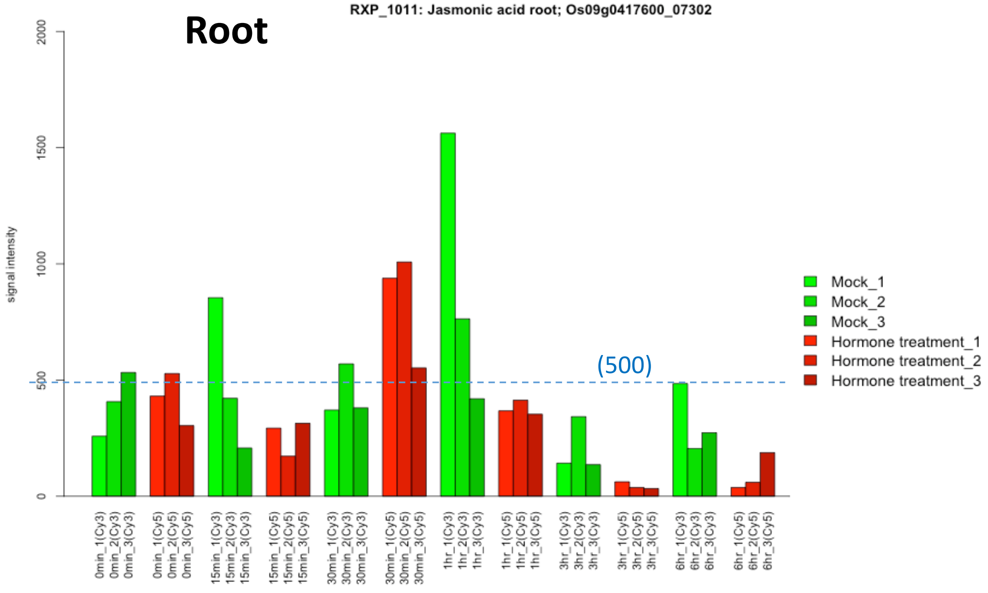

Expression profiles of *OsWRKY76* in shoots and roots after JA treatment. Seven-day-old rice (*Oryza sativa* L. japonica cv. Nipponbare) seedlings were treated with 100 µM jasmonate and RNA expression was observed for 12 hr after jasmonate treatment. The data were obtained from RiceXPro (http://ricexpro.dna.affrc.go.jp/category-select.php).

Supplemental Figure 6

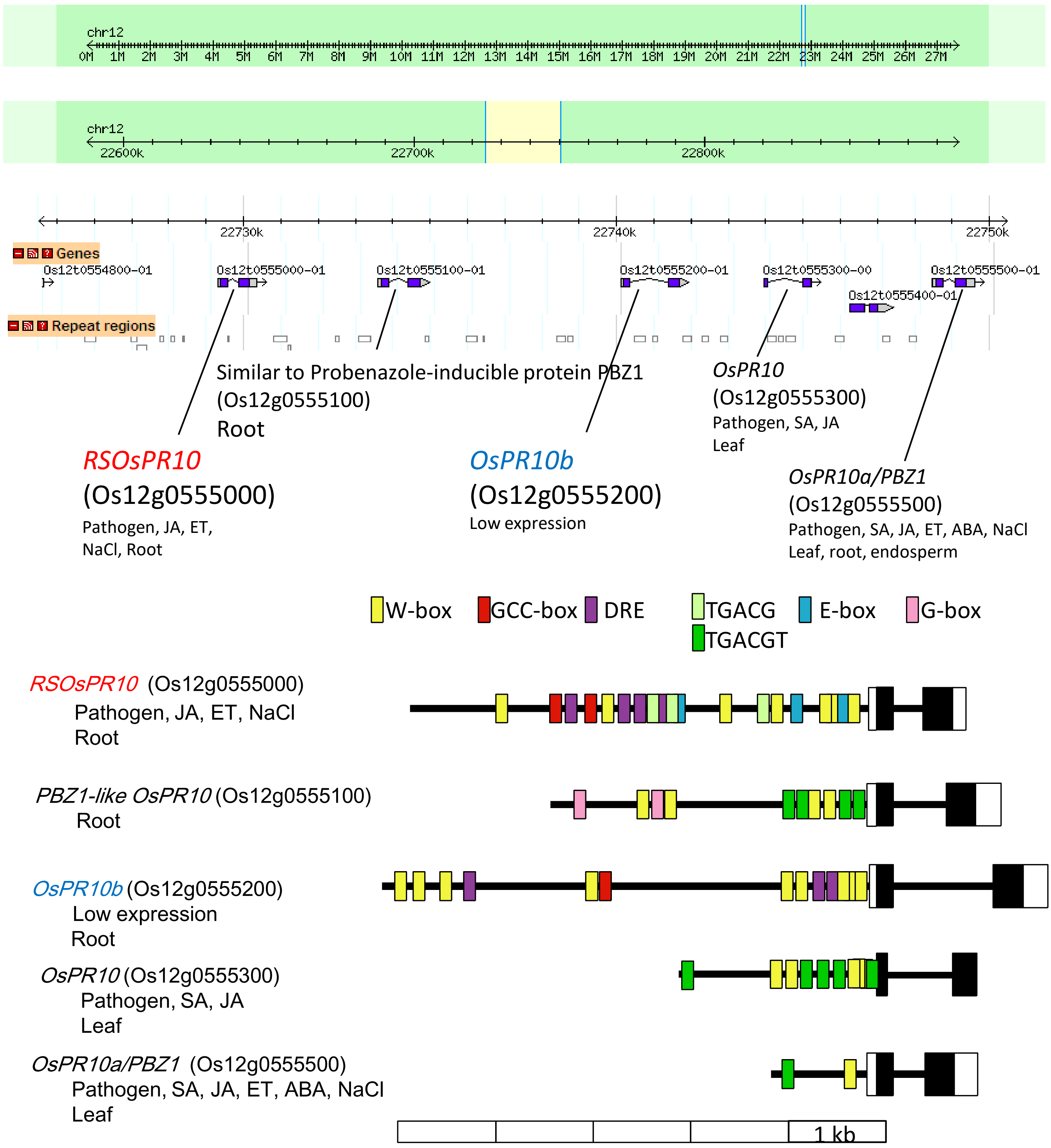

Five rice *PR10* homolog genes, *RSOsPR10, PBZ1-like OsPR10, OsPR10b, OsPR10,* and *OsPR10a/PBZ1*, located in a gene cluster in chromosome 12.
The five genes were induced by pathogen infection, but the expressions were differently regulated by plant hormones and in different organs. *OsPR10a/PBZ1* and *OsPR10* are upregulated by both SA and JA, and *RSOsPR10* and *OsPR10b* expression is induced JA and root specific (http://ricexpro.dna.affrc.go.jp/).

Supplemental Figure 7

RSOsPR10 (Os12g0555000)

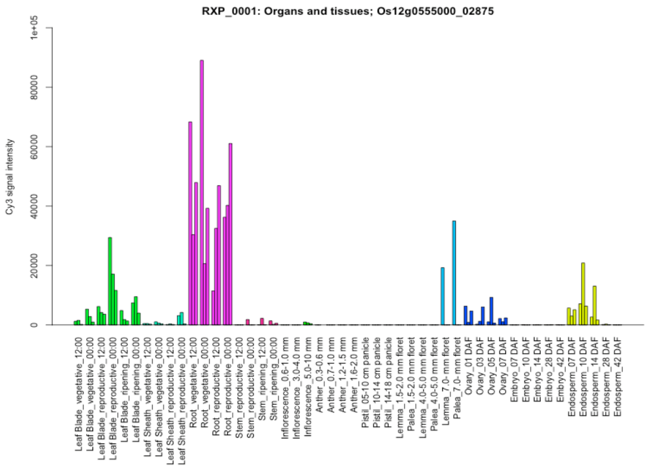

OsPR10b (Os12g0555200)

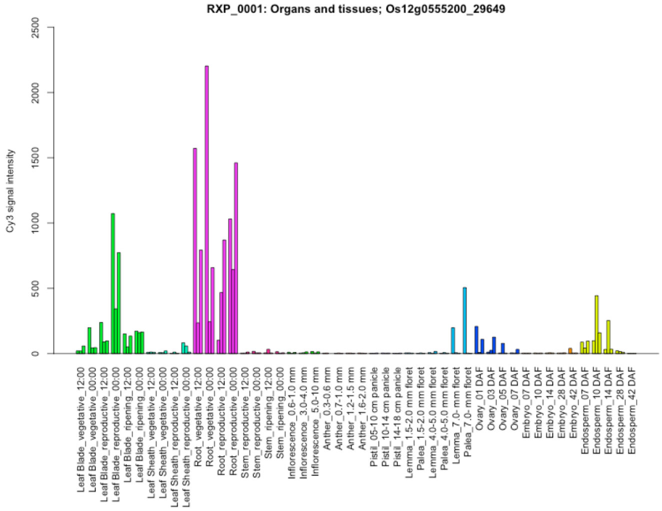

Root

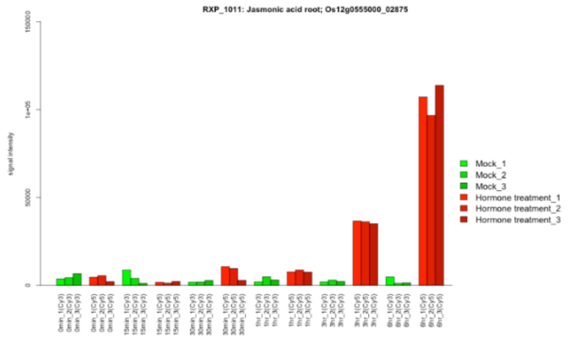

Root

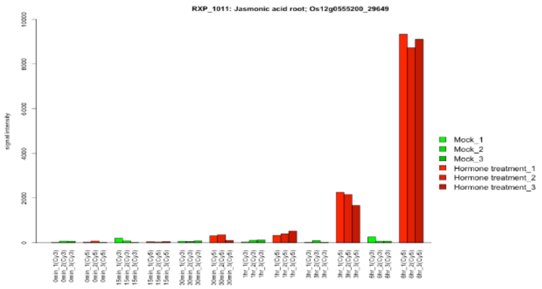

Shoot

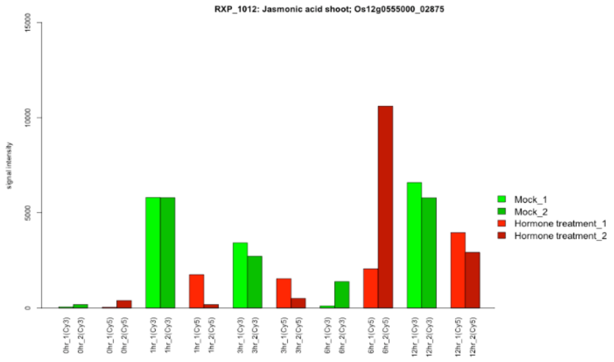

Shoot

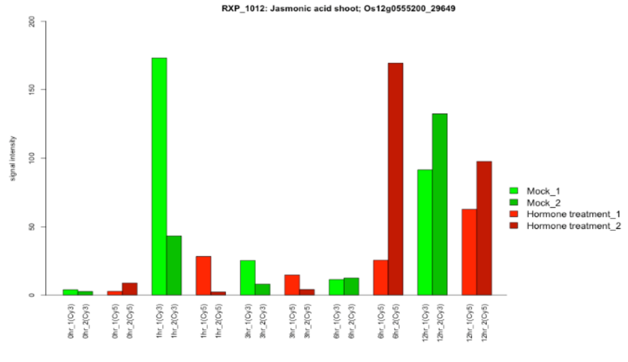

Expression profiles of *RSOsPR10* and *OsPR10b* in rice.
Organ specificity and root specific JA inductivity are very similar, but the expression level of *OsPR10b* expression is much lower than that of RSOsPR10 (http://ricexpro.dna.affrc.go.jp/).

Spatio-temporal gene expression of various tissues/organs throughout entire growth in the field

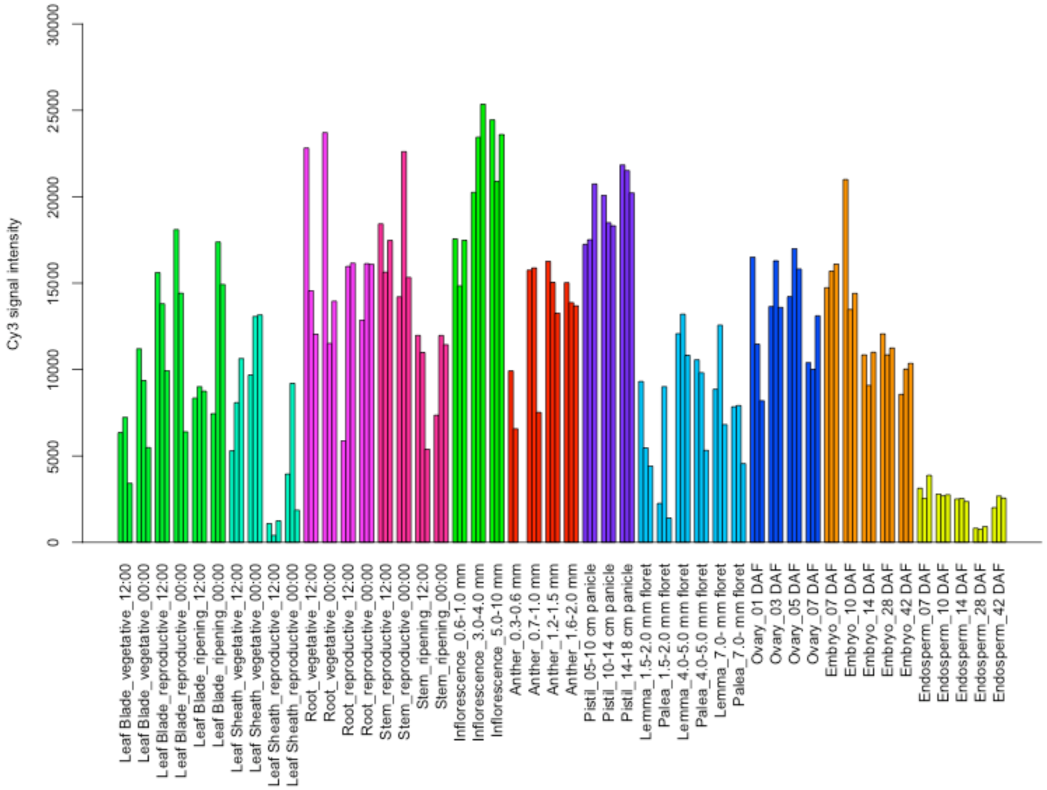

Response to jasmonic acid

Shoot

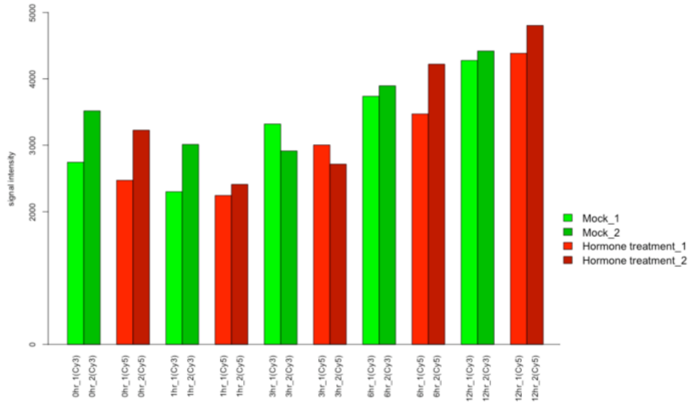

Root

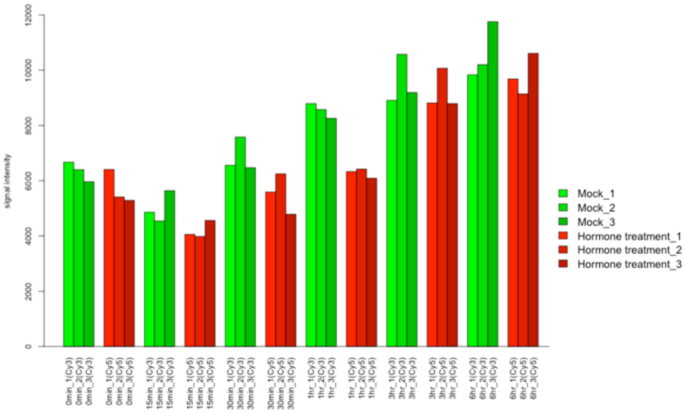

Expression profiles of *MED25* in rice.

Rice MED25（*Os09g0306700)* gene is expressed relatively ubiquitous through plant development and organs. No jasmonate response is observed in shoots and roots (http://ricexpro.dna.affrc.go.jp/).

Supplemental Figure 9

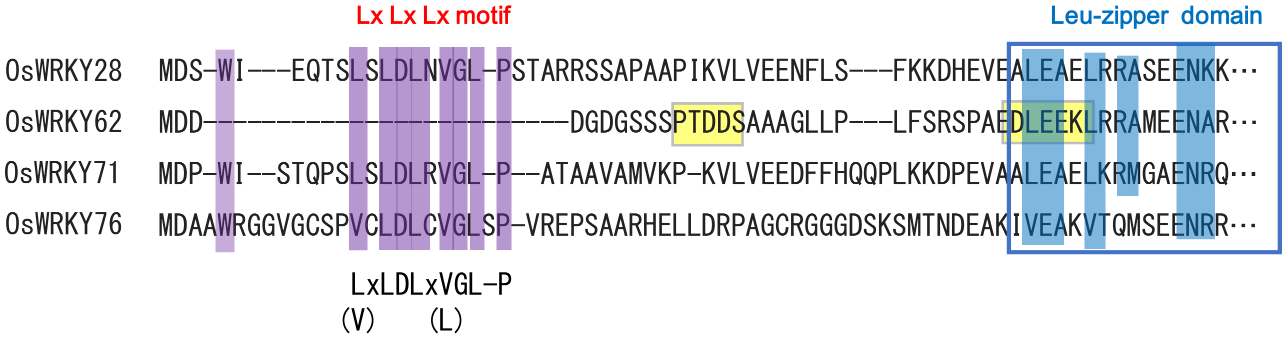

Alignment of amino acid sequence of N-terminal region of four rice type-IIa WRKYs; OsWRKY28, OsWRKY62, OsWRKY71 and OsWRKY76.

These OsWRKYs were reported to have repressor activity. In N-terminal region of OsWRKY28, OsWRKY71 and OsWRKY76, highly conserved amino acid sequence containing LxLxLx putative repressor motif. The sequence is similar to EAR (ethylene response factor associated amphiphilic repression), as reported in some ERFs, AUX/IAAs (Ohta et al., 2001, Plant Cell; Tiwari et al., 2004, Plant Cell; Li et al., 2011, Plant Physiol). Although OsWRKY62 has unique amino acid sequence and no LxLxLx motif in N-terminal region, requirement of TDDS and EDLEEK sequences (highlighted with yellow) for the repressor activity was discussed (Liu et al., 2017 Plant Physiol).
