# Supplementary material for: Expression of RSOsPR10 in rice roots is antagonistically regulated by jasmonate/ethylene and salicylic acid via the activator OsERF87 and the repressor OsWRKY76, respectively
Source: Plant Direct. 2018 Mar 30;2(3):e00049. doi: 10.1002/pld3.49 (PMC6508531; doi:10.1002/pld3.49)
Supplement: Supplementary file 2 [file PLD3-2-e00049-s002.pdf]

Supplemental Table 1

Transcription factors	EAR motif	Reference
Class II ERFs	L/FDLNL/FxP	Ohta et al., 2001 Plant Cell
Zinc finger proteins	L/FDLNLxP	Ohta et al., 2001 Plant Cell
AUX/IAAs	LxLxLPG	Tiwari et al., 2004, Plant Cell<br>Li et al., 2011, Plant Physiol.
NtERF3	LDLNLxP	Tiwari et al., 2004,
AtERF4	LxLNLPP	Tiwari et al., 2004,
AtSUPR	LDLxLxx	Tiwari et al., 2004,

EAR-like LxLxLx putative repressor motif in transcription factors from various plants.
Amino acid sequences of EAR motif in class II ERFs, some zinc finger proteins from various plants, and AUX/IAAs in Arabidopsis were pull out from previous reports (Ohta et al., 2001, Plant Cell; Tiwari et al., 2004, Plant Cell; Li et al., 2011, Plant Physiol).

Supplemental Table 2

Primers used in this study

For cloning to pGEM-T	
forward primer	5'-aagcttATGCAGGTCCAGTTACTGCC-3'
reverse primer	5'-cctaggTGGTGATATATCCCCCACCT-3'.
For GUS constructs	
2K-GUS	5'-tgattacgccaagctAGGCAATACTCCATTCGTC -3'
3K-GUS	5'-tgattacgccaagctGTGCTCCAGCATGCAACGGT -3'
4K-GUS	5'-tgattacgccaagctATGCAGGTCCAGTTACTGCC -3'
reverse primer	5'-ggatccatgttctagTGGTGATATATCCCCCACCT -3'
For LUC constucts	
4K-Fw	5'-ctcggcggccaagctATGCAGGTCCAGTTACTGCC-3'
3.4K-Fw	5'-ctcggcggccaagcttGGGCAACGGATCGACAGT-3'
3K-Fw	5'-ctcggcggccaagctGTGCTCCAGCATGCAACGGT-3'
2.8K-Fw	5'-ctcggcggccaagcttAGCTCCACCCCATCCCTATG-3'
2K-Fw	5'-ctcggcggccaagctAGGCAATACTCCATTCGTCCCA-3'
RSOsPR10-pro-RV-NcoI-C	5'-tttggcatcttccatTGGTGATATATCCCCCACCTGC-3'
For RLUC contruct	
OsUb-pro-pRL-FW	5'-ctcgagctctaagctTGAATCAGCATAGGCTGCCG-3'
OsUb-pro-pRL-RV	5'-agtcatggtggctagCTGCAAGAAATAATCACCAAACAGA -3'
For mutagenesis of GCC- and W-boxes	
Mut-GCC1-FW	5'-TCACGGAGGATAGATTAAGAGGGCCGCT-3'
Mut-GCC1-RV	5'-AAAAATGGATCTTCACGGAGGATAGATT-3'
GCCmut1 forward primer	5'-CAGCGGAGGAACACCTAGTAGCCGGATC-3'
GCCmut1 reverse primer	5'-GGTGTTCCTCCGCTGGGCACCGTTGCA-3'
GCCmut2 forward primer	5'-TCGAGGAGGAGGTGCGCAGGCGGATCGA-3'
GCCmut2 reverse primer	5'-GCACCTCCTCCTCGATCCGGCTACTAG-3'
Mut-GCC2-FW	5'-TCGAGGAGGATGCCATGCTCGGCGGCGG-3'
Mut-GCC2-RV	5'-TGCGCAGGCGGATCGAGGAGGATGCCAT-3'
GCCmut3 forward primer	5'-GCTCGGAGGAGGTTAGATCAGAGGCGAG-3'
GCCmut3 reverse primer	5'-TAACCTCCTCCGAGCATGGCATCCTCC-3'
Mut-W-2.8-F	5'-CGATAGtTCAAGGCGGTGGAGTGCG-3'
Mut-W-2.8-R	5'-GCCTTGAaCTATCGTCGCTGCGAGCC-3'
Cloning of effectors	
OsERF1-Fw	5' -TCTAGATGACGGCGCGAAGCATG-3'
OsERF1-Rv	5' -GAGCTCTTAGATGACGAGCTGCTCCACG-3'
OsERF83-Fw	5' -TCTAGATGCATTGCTGCATGTCGCT-3'
OsERF83-Rv	5' -GAGCTCTCAGATGGAGTGGTGGCTTGG-3'
OsERF87-Fw	5' -GGGGACAAGTTTGTACAAAAAAGCAGGCTCCATGATGCAGCCACCGTACACG-3'
OsERF87-Rv	5' -GGGGACCACTTTGTACAAGAAAGCTGGGTCCTAGGATTGGCAATGACGATTTG-3'
OsERF136-Fw	F: 5'- CACGGGGGACTCTAGATGGAGCAACACAGCCAC-3'
OsERF136-Rv	R:5'- ATCGGGGAAATTCGTCAATGGCTAGTTTGCTCG-3'
For qRT-PCR assay	
GUS forward primer	5'-CTGATAGCGCGTGACAAAAA-3'
GUS reverse primer	5'-GGCACAGCACATCAAAGAGA-3'
UBQ forward primer	5'-TCCGTGGTGGTCAGTAATCA-3'
UBQ reverse primer	5'-ACTGCTGTCCCACAGGAAAC-3'
RSOsPR10-FW5	5' -ATGAAGCTCAACCCTGCTGT-3'
RSOsPR10-RV6	5' -TGAGCTTGCCCACCTTACTT-3'
ERF87-Right-3	5' -GCTCCTCCAGGTAGTCGGC-3'
ERF87-Left-3b	5' -GCAAGCCTACCAAGCACATGC-3'
OsWRKY76-F1	5' -AGAAGAGGAGCAGGGAGAGC-3'
OsWRKY76-R1	5' -CATTGGTACCCGTCCTTGAC-3'
