# Supplementary material for: Expression of RSOsPR10 in rice roots is antagonistically regulated by jasmonate/ethylene and salicylic acid via the activator OsERF87 and the repressor OsWRKY76, respectively
Source: Plant Direct. 2018 Mar 30;2(3):e00049. doi: 10.1002/pld3.49 (PMC6508531; doi:10.1002/pld3.49)
Supplement: Supplementary file 3 [file PLD3-2-e00049-s003.pdf]

February 12, 2018

Manuscript (2017-00113-T1)

Dear
Barbara De Coninck
Ivan Baxter
Editor, Plant Direct

Thank you for your helpful and valuable comments as well as those of the reviewers regarding our manuscript, which you asked to revise it on Jan 28. We appreciate the opportunity to resubmit a revised version of our manuscript. According to your suggestions and the reviewers' comments, we have carefully revised our manuscript, which is now titled, "Antagonistic Regulation of *RSOsPR10* Expression by Jasmonate/Ethylene and Salicylate Pathways is Mediated by OsERF87 Activator and OsWRKY76 Repressor, respectively, in Rice Roots". Our point-by-point responses to all suggestions and comments are provided in the following pages, and corrected parts are highlighted with red characters.

We hope that our revised manuscript adequately addresses all suggestions and comments, and that it is now suitable for publication in *Plant Direct*.

Thank you very much for your help in improving our manuscript. We look forward to hearing from you at your earliest convenience.

Sincerely,

Tomokazu Koshiba

Department of Biological Sciences
Tokyo Metropolitan University
1-1 Minami-Osawa, Hachioji
Tokyo 192-0397, Japan
Tel./Fax: +81 42 677 2565
E-mail: koshiba-tomokazu@tmu.ac.jp

We have made major revisions according to Editor's comments as follows.

Editor:

(1) Please make sure that your statistical tests are correct and (2) as one reviewer also suggested I would definitely adapt the title which is now very confusing. Additionally, please (3) address reviewer #1s points 2-5. (4) I have one additional remark for Figure 1D and E: the color difference between mock and SA should be more clear. The rest of the review comments should be considered excellent suggestions for improving the manuscript, but are not required to meet our acceptance criteria.

(1) > We have corrected the descriptions where we stated lower/higher in a shorter/variant of promoter region, or in a mutant plant.

Lines 200??: "The promoters with single mutations, *3K*[G4m] or *3K*[W2m], resulted in reporter expression levels similar to those obtained with the control *3K* promoter, with JA-induced expression levels almost double those in the mock-treated plants. Fold changes of +JA/mock with *3K*, *3K*[G4m] and *3K*[W2m] were 2.34, 2.37 and 2.21, respectively. However, the expression level of *3K*[G2–G5m] after JA treatment (fold change, 1.97) was lower as that of *2.8K* promoter (fold change, 1.55) (Figure 3C)." (**lines 200-203**)

Lines 286?: The levels of FLUC expression after JA treatment was significantly increased in wild-type and *OsWRKY76-KO* plants, but the level did not change in *OsWRKY76-OX* plants (Figure 6D). (**lines 291-293**)

Figure 6A: Added statistical difference between OsERF87 and OsERF87+OsWRKY76 (**Figure 6A**).

(2) > We have changed the title to "Antagonistic Regulation of *RSOsPR10* Expression by Jasmonate/Ethylene and Salicylate Pathways is Mediated by OsERF87 Activator and OsWRKY76 Repressor, respectively, in Rice Roots". (**lines 1-3**)

(3) Reviewer #1

2, Fig. 5B, Mock treatment is required in the JA treatment experiment.

> We have made additional experiments with mock and +JA, and replaced a new figure (**Figure 5B**).

In Fig. 5C, it is better to show the plant cells in the same sizes.

> We have changed the photos with the same sizes (**Figure 5C**).

3, The authors mentioned that OsWRKY76 knockout plants were obtained by homologous recombination. However, in supplemental figure 4, expression of OsWRKY76 was analyzed. A detail description OsWRKY76 knockout is required.

> We confirmed the disruption of the DNA sequence and the expression of *OsWRKY76*. We have deleted the expression data of O*sWRKY76 KO* rice in the original Supplemental Figure 4, because it causes confusion, and rephrased the description to "The *OsWRKY76* transcript levels in wild-type plants were very low in mock and JA treatment, but apparently induced by SA, while the levels were about 10 times higher in *OsWRKY76-OX* plants than in wild type, even mock and JA treatment (Supplemental Figure 4). No JA inductivity of OsWRKY76 in roots of rice seedlings are also shown [RiceXpro database (http://ricexpro.dna.affrc.go.jp/)] (Supplemental Figure 5).". (**lines 281-286**).

We have also added a detail description of *OsWRKY76 knockout* in the methods section. (**lines 482-491**)

4. Fig. 6B, the quality of EMSA picture is not good enough.

> We have tried to get a better quality of EMSA picture and replaced in Figure 6B.

5. The authors drew two models for OsERF87 and OsWRKY76 regulation of RSOsPR10, in Figure 7. Actually, OsWRKY76 was rapid and strongly induced by wounding and MeJA (Yokotani et al., 2013; Liu et al., 2016). It is not suitable to define OsERF87 only response to JA and OsWRKY76 only to SA.

> We carefully checked the results described by Yokotani et al, JXB 2013 and Liu et al, Plant Physiol 2016 again, and confirmed that they have done only with aerial parts (shoots) of rice seedlings. Also, as shown in Supplemental Fig 5 (in the revised ver.), our result showed no *OsWRKY76* induction by JA, at least 12 h after JA treatment in roots (while, it was induced by SA). In addition, we searched RiceXpro where any induction of *OsWRKY76* by JA treatment is not observed in rice roots (we have added this information in Supplemental Fig 4 (in revised ver.)). And, we have added some description in the text, as "The gene expression of these *OsWRKYs* are known to be up-regulated by blast fungus inoculation and benzothiadiazole (BTH) treatment, and therefore it is probable that the regulation is down-stream of the SA signaling pathway. However, it was also reported that *OsWRKY76* was induced by wounding and MeJA in rice seedlings, indicating the involvement of JA signaling pathway (Yokotani et al., 2013; Liu et al., 2016). These results were obtained with leaves and shoots, but studies with roots have not been done so far. In the present study, we checked the effect of JA and SA treatments on *OsWRKY76* expression in roots of wild type seedlings and the result showed that it was induced by SA treatment but not by JA treatment (Supplemental Figure 4). No response against JA on *OsWRKY76* induction in rice roots was also shown in RiceXpro database (Supplemental Figure 5). Therefore, it is likely that *OsWRKY76* induction by wounding and/or JA is differently regulated in shoots and roots." (**lines 386-397**)

We have also added "in Rice Root" to title of Figure 7.

(4) > We have changed Figure 1D and E: the colors of mock and SA showed the differences more clearly.

Our responses to reviewer's other comments.

Reviewer #1 :

1, P21/424, the references is in compatible with what described in the contest about OsWRKY76 form complexes.

> Thank you for this comment. We made mistake so have changed the sentence; "As reported previously, *Arabidopsis* and rice WRKY proteins in group II and III often form homo- and hetero-dimers and the forms are suggested to affect their transcriptional activity (Chi et al, 2013; Liu et al, 2016)." (**lines 439-441**)

Reviewer #2 :

In the model given in Figure 7B, I wonder if the biotic stress only induce the SA pathway? It is possible that both SA and JA pathways are activated simultaneously by a biotic stress and the cross talk between these two pathways simultaneously regulate the expression of this gene. So, it is not clear to me why the salt inducibility of OsERF87 was shown in the model in Figure 7A as this information is at least partly derived from previous studies (eg Takeuchi et al 2011) and abiotic stress (eg salt) and biotic-abiotic stress crosstalk involved in the regulation of ROsPR10 were not investigated in the paper. Perhaps both JA and SA regulations of ROsPR10 by OsERF87 and OsWRKY76 could be combined into a single model such as the one shown in Figure 7B. As indicated in the last sentence of summary, this mechanism (ie antagonism) represents a fine-tuning system to sense the balance between JA/ET and SA signalling in plants under environmental stress.

> We agree some parts of this comment, but as explain above (reviewee #1-1), at least in roots of rice seedlings, it is reliable that *OsWRKY76* is induced by SA but not by JA. Therefore, we think Figure 7B is okay in this ms, because it is focused on rice roots.

Explain also in the Figure legend what broken lines indicate on this figure.

> We put explanation of broken line in legend of Figure 7.

Line 1. The wording of the title could be improved to make it more meaningful. An alternative suggestion would be "Differential Regulation of RSOsPR10 by Jasmonate/Ethylene and Salicylic Acid Pathways is mediated by OsERF87 Activator and OsWRKY76 Repressor, respectively, in Rice Roots" given that the differential regulation of RSOsPR by JA and SA pathways is already known.
> We have revised the title according this suggestion as explained above.

Line 33 Perhaps use "different promoter::β-glucuronidase (GUS) deletion constructs" instead of "different-length promoter::B-glucuronidase (GUS) constructs'.
> We prefer the latter.

Line 59 Why were the initials of the first author given for this reference (Huang, L.F. 2016)?
> There are two articles by same first name authors with same year (2016). To discriminate these, we put their initials in the text. [Huang, P.Y. et al. 2016] and [Huang, L.F. et al. 2016]

Line 65. "further studies on the roles and functions of PR proteins".. What is the difference between "roles" and "functions" in this sentence?
> We have changed to "physiological and molecular functions". (**line 66**)

Line 76 SA pathways. There is only one SA pathway?
> We have corrected.

NPR1, should the full name be given when it is first used? Check journal regulations.
> We have added the full name of NPR1 "Nonexpressor of pathogenesis-related genes 1". (**lines 77-78**)

Line 82. "Saving energy by controlling the allocation of resources among defense responses to simultaneous biotic and abiotic challenges." I think this is a little misstated and may require rephrasing. This antagonism is considered to be required for allocation of resources depending on the types of attackers (biotrops vs necrotrophs) not for biotic and abiotic stress factors.
> We agree with this statement, so have changed to "depending on the types of attackers". (**line 83**)
However, we think this concept is not so clear and more widely used for antagonistic regulation between different environmental stresses, including both biotic and abiotic stresses. Actually, almost these stresses are mainly transduced via plant hormones, such as ET, JA, SA and ABA, to modulate many defense genes, where the cross-talks between the hormone signaling are attractively studied in recent plant science field. Among them, SA-JA crosstalk has been deeply investigated and discussed their molecular mechanism and

physiological meaning. And one of important questions is “When plants are suffered two (or more) different stresses, how plants survive under limited energy resources?”.

Line 87 "Factors in JA/ET and SA signalling pathways, like COI1, JAZs, NPR1, MYC2, TGAs, ERFs and WRKYs, are known to be attenuators of PDF1.2". I think this needs to be rephrased as well or the word "attenuators" need to be replaced with "regulators". Otherwise, COI1 and some ERFs such as ERF1 and ORA59 are positive regulators of PDF1.2, not "attenuators".

> We have changed to “regulators”. (**line 89**)

Line 91 GCC box rather than GCC elements.

> We used both “box” and “element”, so have changed all to “box(es)” in the text.

Line 236 "likely" instead of "reliably".

> We have changed to “likely”. (**line 240**)

Line 269. Use the long name of MBP "maltose binding protein" above, when it was first referred to MBP in line 266.

> We added full name “maltose binding protein”. (**line 270**)

Line 314 "molecules" or "regulators"?

> We think it better to use “molecules”.

Reviewer #3 :

- The title is hard to understand without reading the paper: What is regulated by what? What is the role of JA/ET and SA here? Do they activate the RSOsPR10 or the ERF87 or the WRKY76 gene?

> We have changed the title as explained above.

Introduction

- In the introduction a lot of literature concering Arabidopsis shoot defence regulation is being described, such as the known regulation of PDF1.2 by antagonistic crosstalk between JA/ET and SA. Since the current research is done on rice, and more specifically on root tissue, the difference/similarity between defence mechanisms in monocots-dicots and roots-shoots should be discussed here.

> Yes, this comment is just one of important issues in plant defense mechanisms, but a body of works have been done with Arabidopsis and still less studies to know difference between monocot-dicot, and roots-shoots. At present, it is quite difficult to discuss about these subjects. We are thus focusing on rice roots, using root specific PR protein, RSOsPR10, in a series of our studies as published by Hashimoto et al., Plant Cell Physiol, 2004, Takeuchi et al., Plant Cell Physiol, 2011, Takeuchi et al., Plant Cell Tiss Organ Cult, 2016, and present study.

- More details should be provided about the role of ERFs. Seeing the JA-based induction pattern of the investigated promoter, it would also be interesting to study the role of MYC transcription factors in activation of this gene. What was the rationale to focus the current study on ERFs, rather than JA-inducible MYCs?

> We thought the same things, but as shown in Fig. 1, there is no MYC binding *cis*-element in RSOsPR10 promoter region. However, OsERF87 could be regulated by OsMYC, or related protein factors, because MYC binding *cis*-elements are found in OsERF87 promoter region. We, therefore, put "OsMYC" in Fig. 7.

Results

- The authors state (page 11, line 188) that the 3K promoter region is important for 'root-specific expression'. However, LUC expression is equally high in mock-treated leaves as in mock-treated roots (Fig. 3B). So, this gene is not root-specific, rather its JA-inducible expression is root-specific.

> We have changed the sentence to "These results indicated that the *3K* promoter region is important for JA induction in the root, consistent with the results obtained using transgenic plants (Figure1)." (**lines 189-190**)

It is also possible that the TFs that activate this gene are root-specific?

> *OsERF87* expression is abundant in the root (about 1-2 order higher than shoots), and JA inductivity is only detected in the root (Please see supplemental Fig. 3).

GUS figures could be provided to demonstrate lack of expression in the leaves.

> Our course of studies clearly showed the expression of RSOsPR10 (both mRNA and protein) is root specific (PCP 2004, PCP 2011, and Fig. 1 in present study). In PCP 2011, we showed no apparent expression of the protein in leaves by immune-histochemical method as well.

- Page 11, line 192 and page 14, line 263: WRKY transcription factors are not strictly regulated by SA. Some WRKYs are also activated by JA-treatment. What is the evidence that OsWRKY76 would be involved in the SA-pathway or would be strictly SA-inducible? According to literature, this gene is also induced by wounding (JA) and ABA (Yokotani et al., 2013).

> As explained as above.

- In the results section, the authors frequently compare the level of induction/repression of this promoter under different conditions. In quite a lot of cases they say things like 'the JA-induced or SA-repressed expression of this promoter region was lower/higher in comparison with ... a shorter/variant of promoter region, or in a mutant plant' (e.g. page 11, line 200; page 15, lines 283 and line 286-287). However, this difference in induction/repression level is never statistically compared between the different regions or mutant forms. In all figures the authors should provide statistical comparison for all possible differences, to proof that the JA-induction or SA-repression is indeed higher or lower in particular cases. I recommend to use letter-coding for each bar in a graph to show all pairwise comparisons.

> As explained as above, we corrected some parts in the text.

- Page 12, line 221-224: are the three ERFs described here (and certainly ERF87) also induced by ET, next to JA?

> We did not investigate about ET inductivity in this study. In general, these factors are induced by ET as studied well in Arabidopsis. Because we showed that RSOsPR10 (both mRNA and protein levels) is induced by ET (ACC) in rice roots (Takeuchi et al., 2011, Plant Cell Physiol), we believe this induction also via OsERF87.

Discussion

- Since this gene is specifically active at the lateral root primordia, I am wondering whether it is regulated by auxin. Can the authors comment on this?

> We agree this comment, so stated in the text, “The results of the present study also showed that the promoter region, at least 3-kb, of *RSOsPR10* drives root-specific gene expression in lateral root primordia and at the basal region of growing lateral roots (Figure 2). Several other studies have suggested that JA and auxin signaling and their crosstalk are important in the formation and growth of lateral roots (Sun et al., 2009; Lu et al., 2015; Ito et al., 2016). Therefore, we hypothesize that RSOsPR10 is one of the key molecules in root development. Further studies on the physiological roles and molecular functions of *RSOsPR10* could reveal mechanisms of root development and the formation of root system architecture, leading to the engineering of stress-tolerant rice.” (lines 315-323)

- Line 315-317: this statement is very vague. How can studying the mode of expression of this gene lead to the formation of stress-tolerant rice?

> We have revised the description to “Further studies on the physiological roles and molecular functions of RSOsPR10 could reveal mechanisms of root development and the formation ….”. (**line 321**)

We mean, for example, increasing RSOsPR10 expression must attain higher tolerance against drought stress, resulting improvement of crop production. In fact, our previous work (TCPOC, 2016) showed RSOsPR10 OE rice and bentgrass conifer tolerance against drought (and salt) stress. In addition, we think difference of root system architecture is important for individual plant growth, and so, if the RSOsPR10 is involved in the regulation of lateral root formation and growth, more detail regulation mechanism and resolution of molecular function of RSOsPR10 are profitable for agricultural application in the future.

- Just like in the introduction, many studies described here (e.g. about the role of ERFs in immunity) were done on Arabidopsis. Can we simply translate this to rice?

> We think it is not easy at present. However, recent accumulating studies show some fundamental mechanisms are very similar among all plant species. It must be good to marge between different plant species, such systems for hormone receptors to gene regulation (e.g. IAA, GA, SA, JA and ET), transcriptional regulation by trans-factors and cis-elements, and photo-receptor systems, so on.

- Page 22, line 443: RSOsPR10 enhances lateral root growth: this statement needs a reference.

> We already refer one of our recent article by Takeuchi et al, 2016 in the first paragraph of Discussion. (lines 309-312)

M&M

- Were plants pooled to have enough material for RNA extraction?

> Yes, we think all samples were enough for RNA extraction.

- A lot of the here-provided data is based on qRT-PCR experiments. In a good qRT-PCR analysis at least 2, preferentially 3, internal reference genes are used for data normalization. In this research, only one was used. How stable is this gene upon the variety of treatments executed here?

> Yes, we did check four genes as internal references (UBQ, Tubulin-alpha, GADPH, APX), and chose UBQ as a stable reference gene under experimental conditions in the present study.

Style/grammar:

- Page 4, line 39-40: I suggest to rephrase to 'Presence of the SA-inducible OsWRKY75 transcription factor strongly repressed/prevented the JA-inducible and OsERF87-dependent expression of RSOsPR10'.

> Thank you for the suggestion. We have rephrased to “In contrast, the SA-inducible OsWRKY76 transcription factor strongly repressed the JA-inducible and OsERF87-dependent expression of *RSOsPR10*.” (**lines 40-41**)

- All abbreviations should be written in full upon first use.

> We have checked throughout the text, and revised with red characters.

- Page 9, line 159-161: please rephrase for clarity.

> We have rephrased to "The ethylene biosynthetic precursor 1-aminocyclopropane-1-carboxylic acid (ACC)-induced *GUS* expression and its suppression by SA were also observed in roots of the *3K::GUS* and *4K::GUS* lines (Figure 1E)". (**lines 161-162**)
